# Bounded rationality in *C. elegans* is explained by circuit-specific normalization in chemosensory pathways

**DOI:** 10.1038/s41467-019-11715-7

**Published:** 2019-08-13

**Authors:** Dror Cohen, Guy Teichman, Meshi Volovich, Yoav Zeevi, Lilach Elbaum, Asaf Madar, Kenway Louie, Dino J. Levy, Oded Rechavi

**Affiliations:** 1 Department of Neurobiology, Wise Faculty of Life Sciences, Tel Aviv University, Tel Aviv-Yafo, Israel; 2 Sagol School of Neuroscience, Tel Aviv University, Tel Aviv-Yafo, Israel; 3 Statistics and Operation Research, Tel Aviv University, Tel Aviv-Yafo, Israel; 4 Center for Neural Science, New York University, New York, NY USA; 5 Coller School of Management, Tel Aviv University, Tel Aviv-Yafo, Israel

**Keywords:** Decision, Economics, Decision making

## Abstract

Rational choice theory assumes optimality in decision-making. Violations of a basic axiom of economic rationality known as “Independence of Irrelevant Alternatives” (IIA) have been demonstrated in both humans and animals and could stem from common neuronal constraints. Here we develop tests for IIA in the nematode *Caenorhabditis elegans*, an animal with only 302 neurons, using olfactory chemotaxis assays. We find that in most cases *C. elegans* make rational decisions. However, by probing multiple neuronal architectures using various choice sets, we show that violations of rationality arise when the circuit of olfactory sensory neurons is asymmetric. We further show that genetic manipulations of the asymmetry between the AWC neurons can make the worm irrational. Last, a context-dependent normalization-based model of value coding and gain control explains how particular neuronal constraints on information coding give rise to irrationality. Thus, we demonstrate that bounded rationality could arise due to basic neuronal constraints.

## Abstract (web summary)

Innate odor preferences in *C. elegans* are controlled by the activation of a pair of olfactory sensory neurons. Here, the authors show that asymmetric activation of the AWC^ON^ and AWC^O^^FF^ neurons can lead to irrational olfactory preferences that are explained by a model of normalization of sensory gain control.

## Introduction

Decision-making is a crucial process that enables organisms to flexibly respond to environmental demands in changing conditions. The choice process has been extensively studied in humans, but it is a general phenomenon extending to the simplest of organisms. A basic assumption in neoclassical theories of choice is that choosers are consistent in their choices. Consistency in choices is the underlying axiom that defines rational behavior^1^.

Normative models of decision-making, such as rational choice theory in economics and foraging theory in ecology, assume optimality in the behavior of individual choosers. Their core principle is utility maximization, which assumes that choosers act to maximize an internal measure of satisfaction. Economists originally proposed that decisions rely only on the outcome probability and magnitude or expected value. However, this simple model fails to describe how human choosers actually behave, leading to the idea that choosers instead transform an expected value into an internal subjective value^2^. While subjective value-based theories can explain behavioral phenomena, such as risk preferences and delay discounting, those normative theories rely on the assumption of consistent choices, and empirical violations of rationality fall outside of their scope.

Humans^3,4^ and other animals^5–11^ can behave irrationally in many contexts. The neuronal mechanisms that lead to irrational behaviors are still unknown. Failures of “rationality”, i.e., inconsistent preferences, may reflect the implementation of decision-making in biological nervous systems facing intrinsic physical and metabolic constraints^12,13^. Despite varying nervous system architectures, all animal tested behave irrationally, suggesting that rationality and deviations from rationality arise from general computational principles rather than specific biological implementations. According to the idea of *bounded rationality*, the computational or informational load required to make truly optimal decisions exceeds the capacity of our nervous systems^12,13^. According to bounded rationality, violations of rationality reflect intrinsic constraints in the decision process, such as limited information, finite decision times, and the inherent limitations of information processing with biological systems. While rationality violations are technically suboptimal according to normative theories, such behavior may nevertheless reflect a more general optimization process: irrational choice behavior could be the cost of a more global optimization over both behavior and neural constraints^14^, or may themselves be favored by natural selection^15^.

One central requirement of rationality and stable value functions is *independence of irrelevant alternatives*, or IIA^16^. According to this axiom, a preference between two options should be unaffected by the number or quality of any additional options, and the relative choice ratio between options A and B (pA/pB) should remain constant regardless of the choice set. However, contextual factors such as choice set size and composition significantly alter animal and human decisions^7,10,17,18^.

To examine the boundaries which lead to irrationality, we established *Caenorhabditis elegans* nematodes as a model organism for rational decision-making. *C. elegans* has only 302 neurons, 32 of which are chemosensory neurons, and uses chemotaxis to achieve sophisticated behaviors, including simple forms of ethologically relevant decision-making^19–23^. Just two pairs of worm amphid sensory neurons, AWC and AWA, are required for chemotaxis toward attractive volatile odors^24^. Specific odors are known to be sensed exclusively by either the AWC or AWA neurons. The two AWC neurons are structurally similar, but functionally distinct from each other and sense different odors^25^. AWC^ON^ detects 2-butanone and acetone, while AWC^OFF^ detects 2,3-pentanedione^25–29^.

In the current study, we examine how *C. elegans* olfactory decision-making depends on the composition of the odorant-defined choice set. We show that in most cases, the worms behave rationally and display consistent choices between two preferred options regardless of the strength of an irrelevant third option. However, asymmetric activation of the AWC neurons by the third odorant can lead to non-optimal decision-making and even preference reversals, which are considered to be “irrational” choices, according to the economic definition of rationality^30,31^. These findings are consistent with a normalization operation in the computation of odor value during decision-making and suggest that specific instances of choice irrationality arise from specific circuit architectures in *C. elegans* value processing and choice behavior.

## Results

### Nonoptimal choice behavior in *C. elegans*

**Fig. 1:**
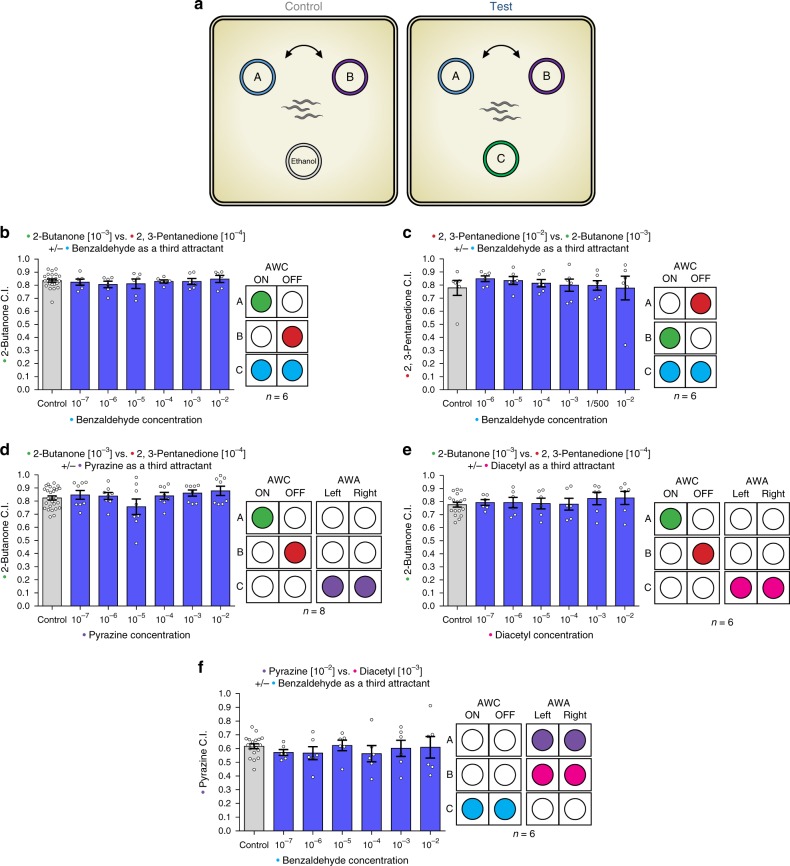
*C. elegans* display rational decisions. **a** A scheme for the Independence of irrelevant alternatives chemotaxis assays. A chemotaxis index (C.I.) (number of worms in A, divided by the number of worms in A and B together) was calculated. Each plate contained ~200–400 worms. **b** The relative preference for 2-butanone over 2,3-pentanedione is unaffected by increasing concentration of benzaldehyde as a third attractant (two-tailed Wilcoxon signed-ranks test; *n* = 6). **c** The relative preference for 2,3-pentanedione over 2-butanone is unaffected by increasing concentration of benzaldehyde as a third attractant (two-tailed Wilcoxon signed-ranks test; *n* = 6). **d**, **e** Introducing AWA- sensed odorants as a third attractant, does not influence the relative preference between 2-butanone and 2,3-pentanedione (two-tailed Wilcoxon signed-ranks test; **d** *n* = 8, **e** *n* = 6). **f** Benzaldehyde as a third attractant, does not affect the relative preference between the two AWA- sensed odorants pyrazine and diacetyl (two-tailed Wilcoxon signed-ranks test; *n* = 6). Two-tailed Wilcoxon signed-ranks test. Bars represent the C.I. of odor A. Error bars represent the standard error of the mean C.I.

To investigate if *C. elegans* exhibits nonoptimal choice behavior, we conducted odor preference tests, as previously described^24,32^. To find “*IIA violations*” we measured the relative preference between two attractant spots, in which the most attractive odor A and less attractive odor B were placed, in the presence or in the absence of a third odor C (Fig. 1a). By changing the concentrations of the odors used in each test, we controlled which specific odorant would be the most attractive (A), the second best (B), and the irrelevant option (C).

We first used odors which are sensed by a minimal decision-making neuronal circuit, by performing choice assays with odors detected exclusively by the two AWC neurons: 2-butanone (odor A), 2,3-pentanedione (odor B), and benzaldehyde (odor C) (Fig. 1b, Supplementary Fig. 1a and Supplementary Fig. 2). In these experiments, the third odor C was sensed by the neurons that sense both odor A and odor B, in a balanced/symmetric way. Namely, since odor C was sensed by both AWC neurons, it could potentially disrupt the sensing of both odor A (sensed only by the AWC^ON^ neuron) and odor B (sensed only by the AWC^OFF^ neuron). We tested the effect that different concentrations of odor C have on the relative preference between A and B. While IIA violations have been observed in a wide variety of organisms^5–11^, despite numerous repetitions and iterations, we found that in *C. elegans* the addition of increasing concentrations of odor C did not lead to violations of rationality, as the preference ratio between odors A and B did not change in a statistically significant or physiologically relevant way. Specifically, in no case did odor B become more attractive relative to A (Fig. 1b and Supplementary Fig. 1a). We performed additional experiments to test the robustness of this rational behavior, and to validate that it does not depend on a specific odor concentration or the identity of odors A and B. The worms still behaved rationally when we changed the concentrations of 2-butanone and 2,3-pentanedione to make 2,3-pentanedione the most attractive odor (A) and 2-butanone the second most attractive odor (B) (Fig. 1c and Supplementary Fig. 1b).

In many organisms, the dopaminergic system has a strong effect on decision-making^33,34^. Therefore, we subjected *cat-2* mutants, defective in dopamine synthesis, to the choice task described above. Similarly to wild-type animals, we did not observe any statistically significant differences in the preference between odors A and B in the presence or in the absence of option C (Supplementary Fig. 3). These experiments suggest that in *C. elegans* the lack of dopamine signaling does not lead to IIA violations.

In the experiments described above, all three odorants (A–C) were sensed by just two neurons, AWC^ON^ and AWC^OFF^. It is possible that this minimal neuronal circuit was “too simple” to give rise to inconsistent behaviors, and irrationality stems from complexity. To increase the complexity of the neuronal circuit underlying the decision process, we tested combinations of odors that are sensed by both AWC and AWA neurons. We started by testing “balanced” third odors C, in the sense that these odors are not sensed preferentially just by the neurons that sense odor A or odor B. We found that increasing the circuit complexity through the addition of another pair of neurons does not lead to inconsistencies in decision-making (Fig. 1d–f and Supplementary Fig. 2c–e). All the results presented above demonstrate that the worm’s decision-making process can be consistent and robust at least when the irrelevant alternative is sensed symmetrically, in a balanced way, by the neurons that sense odor A and the neurons that sense odor B.

**Fig. 2:**
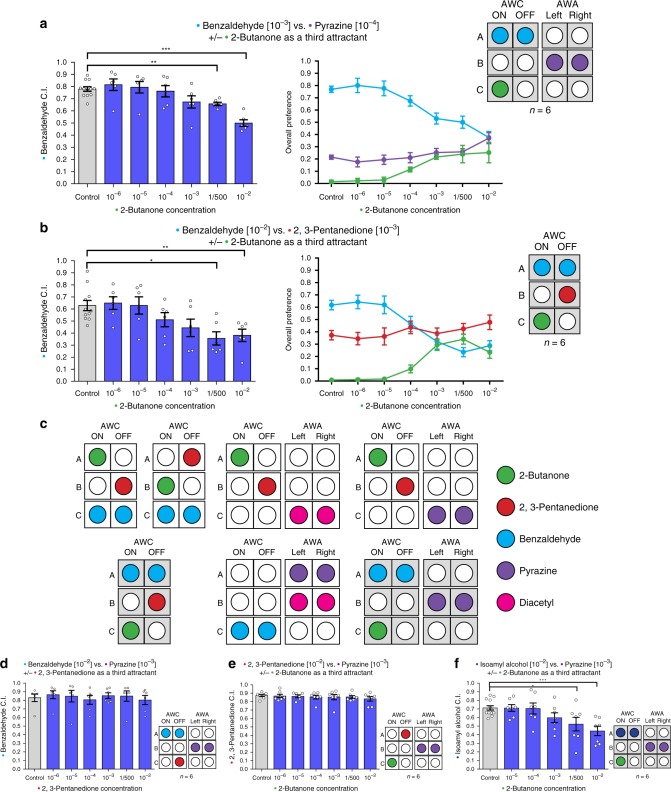
*C. elegans* exhibit IIA violations when specific neuronal architectures are induced. **a** The effect of 2-butanone as a third attractant on the relative preference between benzaldehyde and pyrazine, and the overall preference of each attractant point in every condition (two-tailed Wilcoxon signed-ranks test, *C* = 1/500: *W* = 2, *q* = 0.0012; *C* = 10^−2^: *W* = 0, *q* = 0.0006; *n* = 6). **b** The effect of 2-butanone as a third attractant on the relative preference between benzaldehyde and 2,3-pentanedione, and the overall preference of each attractant point in every condition. (two-tailed Wilcoxon signed-ranks test, *C* = 1/500: *W* = 6, *q* = 0.0144; *C* = 10^−2^: *W* = 2, *q* = 0.0036; *n* = 6). **c** In all the violations that we described so far, 2-butanone, sensed specifically by the AWC^ON^ neuron, functioned as odor C, and benzaldehyde, sensed by both AWC neurons, functioned as odor A. **d** 2,3-pentanedione as a third attractant does not change the relative preference between benzaldehyde and pyrazine (two-tailed Wilcoxon signed-ranks test, *n* = 6). **e** 2-butanone as a third attractant does not change the relative preference between 2,3-pentanedione and pyrazine (two-tailed Wilcoxon signed-ranks test, *n* = 6). **f** 2-butanone as a third attractant significantly reduced the relative preference for isoamyl-alcohol over pyrazine (two-tailed Wilcoxon signed-ranks test, *C* = 1/500: *W* = 14, *q* = 0.0278; *C* = 10^−2^: *W* = 2, *q* = 0.0005; *n* = 7). Bars represent the C.I. of odor A. Error bars represent the standard error of the mean C.I. *q < 0.05, **q < 0.01, ***q < 0.001, ****q < 0.0001

Next, we broke the symmetrical pattern of olfactory inputs, to test if asymmetry in the sensing of the different odors can lead to irrational decision-making. In several experiments testing different sets of odors, we found that IIA violations, as well as preference reversals, can occur due to an *asymmetric overlap* between odors A and C, independently of the number of neurons involved (Fig. 2a, b). More specifically, we found that IIA violations can occur when odor C is sensed in an imbalanced manner by the neurons that sense odor A but not odor B. To our knowledge, this is the first demonstration of economic irrationality and IIA violations in *C. elegans*.

To test whether these conclusions are robust beyond the particular experimental setup, we performed experiments where we ensured that the distance between all three odors and the boundaries of the plate are the same. To do so we used round plates instead of square plates. These experiments showed also that the distance between odor focal points and the radius of the odor focal points do not affect the results (Supplementary Fig. 4). Moreover, to avoid any possible effects of distances, plates sizes, or shapes, we designed a simplified assay, where the worms faced binary choices between odors A and B, and odor C was embedded in the agar plate (see Methods). Again, we observed an IIA violation when we used the same combination of odors (as in Fig. 2a, b) that induces an *asymmetric overlap* between odors A and C (Supplementary Fig. 5). Thus, the specific neuronal architecture involved in odor sensation, and not the experimental setup, determines whether the worms would behave rationally or not.

In all the violations that we have described so far, 2-butanone, sensed specifically by the AWC^ON^ neuron, functioned as odor C, and benzaldehyde, sensed by both AWC neurons, functioned as odor A. Thus, an alternative explanation to these results is that the violations do not occur from breaking of the symmetry in the odors’ sensation but arise instead from a specific interaction between 2-butanone (odor C) and benzaldehyde (odor A) (Fig. 2c). When 2,3-pentanedione (an odor sensed by AWC^OFF^) served as odor C instead of 2-butanone, the worms behaved rationally (Fig. 2d and Supplementary Fig. 6a). It was previously reported that the asymmetry between the two AWC neurons is required for the ability to discriminate between benzaldehyde and 2-butanone. The authors hypothesized that 2-butanone can attenuate benzaldehyde signaling in the AWC^ON^ neuron^28^. Therefore, we conducted different experiments to test if the observed IIA violations stem from specific interactions between 2-butanone and benzaldehyde in the AWC^ON^ neuron. Four lines of evidence suggest that this is not the case, and instead, a general circuit principle of asymmetry in sensation underlies irrationality.

First, when 2-butanone served as either odor A or B, and benzaldehyde served as odor C, no violations were observed (see Fig. 1b, c, Supplementary Fig. 1 and Supplementary Fig. 2a, b). Thus, the worms do not make inconsistent decisions simply because they cannot distinguish between these two odors.

Second, using 2-butanone as odor C is not enough to make the worms irrational; when 2-butanone serves as odor C, but the circuit was symmetrical, the worms made consistent, rational decisions and did not show IIA violations (Fig. 2e and Supplementary Fig. 6b). This shows that 2-butanone cannot be considered as a general “distractor” or “confusant” molecule like the repellent DEET pesticide^35^.

Third, while both benzaldehyde and 2-butanone are attractive odors when presented separately^24^, little is known about the ecology of *C. elegans*^24,36,37^, and it is possible that their combination in the wild is associated with an unattractive or even repulsive substance. In this case, it would be rational for the worm to avoid an unattractive odor, formed by the combination of benzaldehyde and 2-butanone, when both are present on the same plate—it would be a “feature”, not a “bug”. To test this possibility, we examined if worms prefer benzaldehyde over a mixed combination of benzaldehyde and 2-butanone (see Methods). We found that the combination of 2-butanone and benzaldehyde was more attractive than benzaldehyde alone. The spot that contained both odorants was as attractive as would be expected based on the simple summation of the attractiveness of each of the odors alone (Supplementary Fig. 7). Thus, introducing 2-butanone does not create a new unattractive odor (with benzaldehyde) which  could explain the IIA violation that we observed. These results strengthen the hypothesis that the IIA violations occur due to constraints on the neural system—that is, it’s a “bug”, not a “feature”.

Fourth, we found that IIA violations arise also due to exposure to other odors, not just benzaldehyde or 2-butanone, when odor C is sensed asymmetrically by the neurons that sense odor A but not by the neurons that sense odor B. When isoamyl-alcohol was used as odor A instead of benzaldehyde (the two chemicals are sensed by both AWC neurons) and odor C was sensed asymmetrically, we observed an IIA violation as well as irrationality (preference reversal) (Fig. 2f and Supplementary Fig. 6c). Furthermore, when acetone was used as odor C instead of 2-butanone (both are sensed only by the AWC^ON^ neuron) we observed an IIA violation and irrationality (preference reversal) (Supplementary Fig. 8).

In summary, the violations of rationality that we documented do not arise exclusively because of the identity of the two odorants benzaldehyde and 2-butanone but stem from a general property of the asymmetry in the sensation of the odor choices (see Fig. 2 and Supplementary Fig. 6). Our data raised the possibility that violations occur only when odors A and C are both sensed by the same neuron.

To test this possibility, we used mutants which have two AWC^ON^ neurons (AWC^ON/ON^, loss of AWC asymmetry). NSY-1 (Neural SYmmetry) is a mitogen-activated protein kinase kinase kinase  which is required for the asymmetric differentiation of the AWC neurons^28^. Nematodes carrying the *nsy-1(ky542)* allele were shown to have an additional AWC^ON^ neuron instead of the AWC^OFF^ neuron^28^. As expected, these mutants do not perform chemotaxis toward 2,3-pentanedione and are hypersensitive to 2-butanone^28^.

**Fig. 3:**
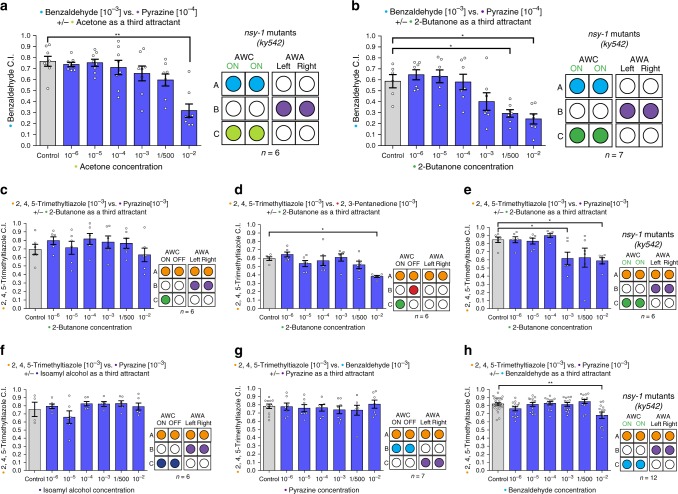
The AWC^ON^ neuron makes the worm vulnerable to IIA violations. **a** The influence of acetone as a third attractant on the relative preference between benzaldehyde and pyrazine, in AWC^ON/ON^ mutant worms. (two-tailed Wilcoxon signed-ranks test, *C* = 10^−2^: *W* = 1, *q* = 0.0018; *n* = 6). **b** The influence of 2-butanone as a third attractant on the relative preference between benzaldehyde and pyrazine, in AWC^ON^/^ON^ mutant worms. (two-tailed Wilcoxon signed-ranks test, *C* = 1/500: *W* = 2, *q* = 0.0141; *C* = 10^−2^: *W* = 2, *q* = 0.0141; *n* = 7). **c** 2-butanone as a third attractant does not change the relative preference between 2,4,5-trimethylthiazole and pyrazine (two-tailed Wilcoxon signed-ranks test, *n* = 6). **d** The influence of 2-butanone as a third attractant on the relative preference between 2,4,5-trimethylthiazole and 2,3-pentanedione (two-tailed Wilcoxon signed-ranks test, *C* = 10^−^^2^: *W* = 0, *q* = 0.0258; *n* = 6). **e** 2-butanone as a third attractant changes the relative preference between 2,4,5-trimethylthiazole and pyrazine in AWC^ON^/^ON^ mutant worms (two-tailed Wilcoxon signed-ranks test, *C* = 10^−2^: *W* = 3, *q* = 0.0456; *C* = 1/500: *W* = 7, *q* = 0.1862; *C* = 10^−3^: *W* = 0, *q* = 0.0132; *n* = 6). **f** Isoamyl-alcohol as a third attractant does not change the relative preference between 2,4,5-trimethylthiazole and pyrazine (two-tailed Wilcoxon signed-ranks test, *n* = 6). **g** Pyrazine as a third attractant does not change the relative preference between 2,4,5-trimethylthiazole and benzaldehyde (two-tailed Wilcoxon signed-ranks test, *n* = 7). **h** Benzaldehyde as a third attractant changes the relative preference between 2,4,5-trimethylthiazole and pyrazine (two-tailed Wilcoxon signed-ranks test, *C* = 10^−2^: *W* = 60, *q* = 0.003; *n* = 12). Bars represent the C.I. of odor A. Error bars represent the standard error of the mean C.I. *q < 0.05, **q < 0.01, ***q < 0.001, and ****q < 0.0001

If inconsistent decision-making stems from interference between two odors sensed by the same neuron, then having the interference occur in more neurons should increase irrational behavior. Therefore, we tested if mutants which have two AWC^ON^ neurons (AWC^ON/ON^) would be more prone to inconsistent decision-making when both odors A and C are sensed by AWC^ON^. When the AWC^ON^-sensed odor acetone was used as odor C, AWC^ON/ON^ mutants made more inconsistent decisions in comparison to wild-type worms and showed irrationality (preference reversal between odors A and B) at lower concentrations of odor C compared to the wild-type worms (Fig. 3a, Supplementary Fig. 9a and Supplementary Fig. 8). As with acetone, the “distracting” effect of 2-butanone, when used as odor C, was much stronger in AWC^ON/ON^ mutants compared to wild-type worms, and irrationality (preference reversal) occurred in lower concentrations of odor C (Fig. 3b, Supplementary Fig. 9b and Fig. 2a).

Note that the AWC^ON/ON^ mutants are hypersensitive to acetone and 2-butanone since they sense these odors using two AWC^ON^ neurons instead of one. Therefore, in relatively low concentrations, these odors instantly became the most attractive odors on the plate (Supplementary Fig. 9a, b). However, this does not change the conclusions of our findings, since an IIA violation is defined as a change in the relative preference between odors A and B^16^.

Following these results, we hypothesized that irrationality arises due to interference of odor C with the sensing of odor A. As noted above, in AWC^ON/ON^ mutants, 2-butanone interferes with the sensing of benzaldehyde in the AWC^ON^ neuron^28^. We examined whether acetone, which like 2-butanone induces irrationality and is sensed only by the AWC^ON^ neuron, also disturbs the sensing of benzaldehyde in the AWC^ON^ neuron. Indeed, we found that in AWC^ON/ON^ mutants, acetone interferes with benzaldehyde sensation (Supplementary Fig. 10). Together, all these experiments support the hypothesis that a neuronal overlap in the sensation of multiple competing odors can lead to IIA violations in olfactory choice behaviors.

In cases where irrational behavior occurs due to interference in the AWC^ON^ neuron, “diluting” the role of the AWC^ON^ neuron in the sensation of odor A should “buffer” against AWC^ON^-dependent irrationality. To test this, we used 2,4,5-trimethylthiazole, which is sensed by both the AWC and AWA neurons^24,25^, as odor A, instead of benzaldehyde or isoamyl-alcohol, which are sensed only by the two AWC neurons. In this setup, the AWC^ON^ neuron comprises only 25% of the neurons that sense odor A, instead of the usual 50%. We examined two odor setups in which the role of AWC^ON^ in the sensation of odor A was reduced, and we did not observe consistent IIA violations in those setups (Fig. 3c, d and Supplementary Fig. 9c, d). We then used the same experimental setup with AWC^ON/ON^ mutants, in which the AWC^ON^ neurons once again surmise 50% of the neurons that sense odor A. When the role of the AWC^ON^ neuron was re-increased, we did find strong changes in the preference ratio of A vs. B (Fig. 3e and Supplementary Fig. 9e). These experiments suggest that the relative weight of the neuron in which the interference occurs in the sensation of odor A affects the tendency to demonstrate inconsistent behavior.

Next, in addition to examining the role of interference in the neuron that senses odor A, we examined the role of the neurons that sense odor C. If inconsistent decision-making stems from interference between odors A and C in the same neuron, then “diluting” the role of this neuron in sensation of odor C should make irrational behavior less likely. To test this, we “expanded” the neuronal circuit that senses the odors (namely involved more neurons in sensation), to dilute the role of AWC^ON^ in the sensation of odor C, while preserving the proportion of neurons that sense each odor. Each of the odors was sensed by twice as many neurons (in comparison to the experiment described in Fig. 2b). While in the previous experiments that showed a violation odor C was sensed solely by AWC^ON^, in this setup the AWC^ON^ only comprised 50% of the neurons that sense odor C. In these experiments, when odor C was sensed also by the AWC^OFF^ neuron, we did not observe any IIA violations, nor did we see any significant changes in the preference of A over B (Fig. 3f and Supplementary Fig. 9f). Similarly, when odor C was sensed by the AWA neurons, we did not observe any IIA violations, nor did we see any significant changes in the preference of A over B (Fig. 3g and Supplementary Fig. 9g). We then performed an experiment in this “expanded circuit” setup with AWC^ON/ON^ mutants, in which the AWC^ON^ neurons are once again the only neurons sensing odor C. When the role of the AWC^ON^ neurons was re-expanded to comprise 100% of the neurons sensing odor C, we observed a significant IIA violation (Fig. 3h and Supplementary Fig. 9h). Thus, the AWC^ON^ neuron—and its relative involvement in representing odors A and C—contributes specifically to the capacity for rational choice behavior in this paradigm.

Next, we examined whether the worms would show IIA violations when odors A and B share a similar set of neurons. We used benzaldehyde (AWC^BOTH^) and isoamyl-alcohol (AWC^BOTH^) as odors A and B, and 2-butanone (AWC^ON^) as odor C. In this setup, when benzaldehyde was odor A and isoamyl-alcohol was odor B we observed a significant violation and even irrationality (preference reversal) (Supplementary Fig. 11a). However, when isoamyl-alcohol was odor A and benzaldehyde was odor B, the worms behaved consistently (Supplementary Fig. 11b). We hypothesized that sensation of benzaldehyde is more dependent on the sensing neuron (in this case AWC^ON^) in comparison to isoamyl-alcohol. To test this hypothesis, we examined the binary preference between benzaldehyde and isoamyl-alcohol in wild-type worms and in AWC^ON/ON^ mutants. We observed that AWC^ON/ON^ mutants have a significantly higher preference for benzaldehyde compared to the preference of wild-type worms (Supplementary Fig. 11c). Thus, the AWC^ON^ is more important for the sensation of benzaldehyde compared to isoamyl-alcohol, perhaps making the worm more prone to irrational behavior when sensing benzaldehyde (see more below).

**Fig. 4:**
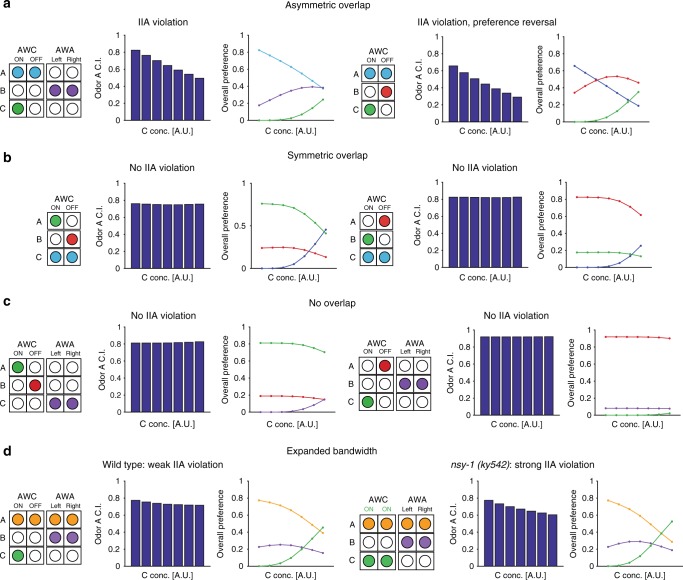
Sensory gain control model of chemosensation explains circuit architecture-specific IIA violations. Predicted choice behavior in a divisive normalization model of sensory gain control in *C. elegans* chemosensation. Odors driving the same chemosensory neuron are assumed to drive cross-odor normalization in neural representation. Simulation parameters were not fit to empirical choice data, but instead were chosen to demonstrate qualitative similarity in behavioral data under different circuit activation patterns. Each combination was simulated for *n* = 10^6^ repetitions. In each combination, the left panel shows the circuit activation pattern, the middle shows the preference index for odor A (relative choice of odors A vs. B), and the right shows the model-predicted preference for the odors. Our results indicate that IIA violations can occur due to an *asymmetric overlap* between odors “A” and “C”. **a** Model-predicted IIA violations in asymmetric overlap circuit architectures. When odors A and C both activate AWC^ON^, increasing concentrations of odor C reduce the representation of odor A in the model via cross-odor normalization. The model captures IIA violations with (right) and without (left) preference reversals, both of which are observed in the empirical data. **b** Model-predicted rational choice behavior in symmetric overlap circuit architectures. In symmetric circuits where odor C activates neurons sensing both odor A and odor B, cross-odor normalization affects the neural representations of both high-value odors. Increasing concentrations of odor C affects the neural representation of odors A and B similarly, and relative preference does not vary. **c** Model-predicted rational choice behavior in nonoverlap circuit architectures. In these circuits, odors A and C activate distinct chemosensory neurons and no cross-odor normalization occurs in the model. Thus, the neural representations of odors A and B (and the relative choice preference of A over B) do not vary with the concentration of odor C. **d** Model behavior in expanded-bandwidth circuits. In expanded-bandwidth scenarios, odor A activates both AWC and both AWA neurons. In wild-type worms, cross-odor normalization only affects 25% of the neural representation of odor A and the model predicts weak IIA violations (left). In *nsy-1* mutants with two AWC^ON^ neurons, cross-odor normalization affects 50% of the representation of odor A and the model predicts stronger IIA violations (right)

Given our behavioral results, we propose a model of pathway-specific sensory gain control and examine its predictions (Fig. 4). The essential feature of the model is that at least some neurons in the chemosensory pathway perform a type of sensory gain control analogous to divisive normalization (see Methods). Critically, cross-odorant gain control only occurs when a given neuron is sensitive to more than one odor and both those odors are present at the same time (i.e., in the same choice set). IIA violations are predicted to occur in scenarios when odor C representations overlap with those of odor A but not odor B (asymmetric overlap). Specifically, increasing concentrations of odor C will divisively scale responses to odor A (when A and B are fixed). Odor B representations, being independent of odor C coding, are unaffected by concentrations of C. Thus, the general prediction is that increasing C will decrease the relative preference of A over B.

Our simulations show that the model predicts empirically observed IIA violations in asymmetric overlap scenarios (Fig. 4a), capturing the decrease in relative preference of A vs. B as C increases. Furthermore, the model can also capture two additional aspects of observed choice: (1) preference reversal of odors A and B, and (2) eventual selection of odor C over odor A. The gain control model also explains why *C. elegans* display rational choice in other circuit activation scenarios (Fig. 4b, c). In symmetric overlap scenarios (Fig. 4b), odor C activates neurons representing both odor A and odor B. Due to symmetric cross-odor normalization in the representations of odors A and B, increasing concentrations of odor C affect both of the computed values of odors A and B equally and relative preference between A and B remains stable. In no-overlap scenarios (Fig. 4c), odor C is sensed by different chemosensory neurons than those sensing odors A and B. In the model, this translates into the equations for the computed value of odors A and B (*R*_*A*_ and *R*_*B*_; see Methods) carrying no C-related terms in the divisive denominator: the activity representing A and B—and thus the relative preference between the two—is independent of distractor odor C.

With additional circuit-specific clarification, the gain control model explains observed choice behavior in expanded-bandwidth scenarios, where the sensing of odor A involves all four chemosensory neurons (Fig. 3 and Supplementary Fig. 9). The gain control model captures this behavior by positing that cross-odor gain control is combined with a weighted sum of representations in all chemosensory neurons detecting a particular odor. Thus, when odor C activates only AWC^ON^, an odor A (e.g., 2,4,5-trimethylthiazole) sensed by all four chemosensory neurons exhibits gain control effects in only 25% of its representation, while an odor sensed by only AWC^ON^ and AWC^OFF^ (e.g., benzaldehyde) exhibits gain control in 50% of its representation; the model thus predicts a diminished effect of contextual odors on choice behavior in expanded-bandwidth scenarios (Fig. 2a and Fig. 3c). Furthermore, consistent with the data, the model predicts (Fig. 4d) that AWC^ON/ON^ mutants—which exhibit the equivalent of two functional AWC^ON^ neurons—should exhibit stronger IIA violations than wild-type worms in identical choice conditions (e.g., when odor C drives cross-normalization in AWC^ON^ neurons).

The principal model features—cross-odor normalization and weighted summation of chemosensory neuron activity—also address a key issue raised by the empirical data. The lack of IIA violations in symmetric overlap scenarios—when odor C drives neurons that represent both odor A and odor B (Fig. 1b, c)—suggests that gain control occurs in both AWC neurons. Under the normalization model, this occurs because cross-normalization in both AWC neurons affects the representations of odors A and B similarly (Fig. 4b). However, the empirical data also suggest a particularly important role for the AWC^ON^ neuron: most of the observed IIA violations involve odor C activation of AWC^ON^ (Fig. 2a, b, f and Fig. 3a, b), worms display rational choice when odor C drives AWC^OFF^ alone (when odor A is benzaldehyde; Fig. 2d), and AWC^ON/ON^ mutants show enhanced irrationality (Fig. 3e). What explains the particular contribution of AWC^ON^ if cross-odor normalization occurs in both AWC neurons? We hypothesize that in the weighted summation of chemosensory neuron activations to compute odor value, there is a relative overweighting of AWC^ON^ vs. AWC^OFF^ output (specifically for benzaldehyde). Under this assumption, the normalization model reproduces the strong IIA violation with odor C AWC^ON^ activation (Fig. 2a) and the lack of violation with odor C AWC^OFF^ violation (Fig. 2d). Furthermore, a strong biased AWC^ON^ weighting for benzaldehyde (and a relatively unbiased weighting for isoamyl-alcohol) also captures (Fig. S12) the opposite effects of 2-butanone as odor C on benzaldehyde and isoamyl-alcohol (Supplementary Fig. 11a, b) as well as the difference in binary preference between these two odors in wild-type vs. AWC^ON/ON^ mutants (Supplementary Fig. 11c).

**Fig. 5:**
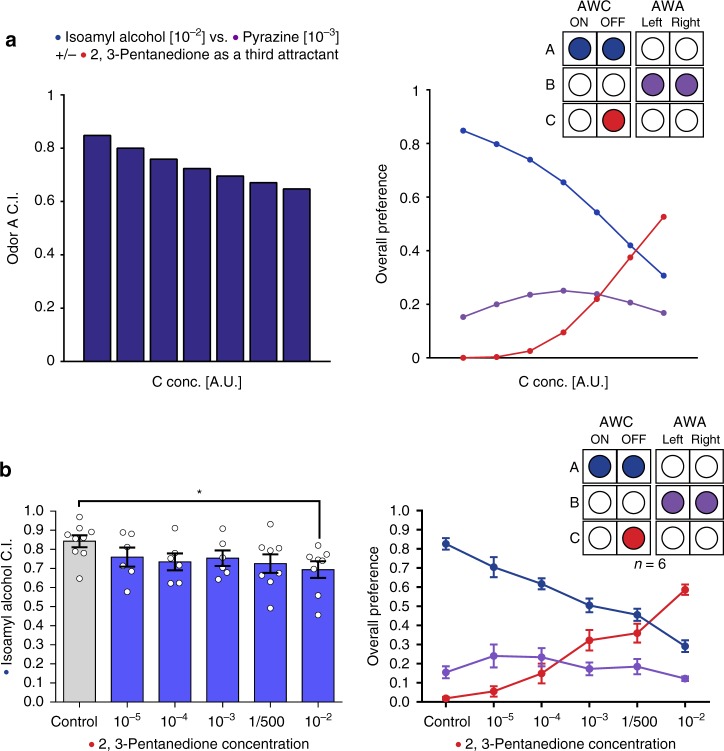
Increasing concentrations of 2,3-pentanedione (AWC^OFF^-sensed odor) as a third alternative can induce IIA violations. **a** Model-predicted IIA violation driven by AWC^OFF^ neuron activation by odor C. Left panel: predicted preference index for odor A (isoamyl-alcohol) relative to odor B (pyrazine) at different concentrations of odor C (2,3-pentanedione). Right panel: predicted preference for all three odors. In the model, the ability of the AWC^OFF^ neuron to generate IIA violations arises from cross-odor normalization and an equal weighting of AWC^ON^ and AWC^OFF^ neuron activity in the representation of odor A (isoamyl-alcohol). **b** The influence of 2,3-pentanedione (AWC^OFF^) as a third attractant on the relative preference between isoamyl-alcohol (10^−2^) (AWC^BOTH^) and pyrazine (10^−3^) (AWA) (two-tailed Wilcoxon signed-ranks test, *C* = 10^−5^: *W* = 17, *q* = 0.2721; *C* = 10^−4^: *W* = 12, *q* = 0.1424; *C* = 10^−3^: *W* = 13, *q* = 0.1424; *C* = 1/500: *W* = 19, *q* = 0. 0.1424; *C* = 10^−2^: *W* = 8, *q* = 0.0275; *n* = 6). Bars represent the C.I. of odor A. Error bars represent the standard error of the mean C.I. *q < 0.05, **q < 0.01, ***q < 0.001, and ****q < 0.0001

Finally, our mathematical model predicts that sensory gain control extends to the AWC^OFF^ neuron. However, all of the IIA violations described thus far are AWC^ON^-dependent. Therefore, we hypothesized that an IIA violation will also arise when the interference between odors A and C occurs in the AWC^OFF^ neuron, specifically for an odor A with relatively equal weighting of AWC^ON^ and AWC^OFF^ representations, e.g., isoamyl-alcohol. According to the model’s predictions, when odor A is isoamyl-alcohol and odor C is 2,3-pentanedione, a weak IIA violation should occur (Fig. 5a). Therefore, we tested this odor combination experimentally, and as predicted by the model, we observed an IIA violation (Fig. 5b). This suggests that the irrational behavior of the worms does not originate from a special trait of the AWC^ON^ neuron, but is a general phenomenon, which stems from the interference between two odors sensed by the same neuron.

## Discussion

In this work, we demonstrate for the first time that even *C. elegans*, with its extremely minimal nervous system, displays IIA violations in decision-making. In most cases, worms behave rationally. However, IIA violations can occur when the different options are represented in an imbalanced way. This imbalanced representation of odors in the AWC neurons interferes with the sensation of the more preferred odor A, thereby decreasing the relative preference for A over B and potentially inducing an IIA violation. We have shown that increasing or decreasing the number of neurons in which the interference occurs, and the relative role they partake in the sensation of odors A and C, can strengthen or diminish the magnitude of IIA violations respectively. The experiments in this paper support the notion that rational behavior in *C. elegans* is determined by the neuronal architecture involved in the sensation of odors, and not by the identity of specific odors.

The fact that an extremely simple organism with only 302 neurons displays IIA violations suggests that nonoptimal behavior originates in basic neural mechanisms common to many species. Moreover, these neural mechanisms directly relate to the high-level descriptions that are commonly used to explain nonoptimal behavior in general, and IIA violations in particular, such as “cognitive overload” or “biased attention”.

The worm is able to distinguish between different odors efficiently, presumably by using sophisticated signal transduction machinery^38^. Expanding the variety of recognizable odors may have come at the expense of consistent decision-making, which may lead to nonoptimal decision processes. Indeed, in *C. elegans*, a single neuron can express many different chemoreceptor genes^39–41^. Evolution may, therefore, have adopted a pattern of increased biochemical complexity to compensate for the lack of neural plasticity and the diminished neuroanatomical complexity in the nematode nervous system^38,42^. These constraints may render such tradeoffs between olfactory repertoire and decision optimality profitable.

IIA violations could also stem from environmental information available to *C. elegans* in the wild. Since we lack information about the ecology of the worm, we as observers may be unaware of information such as the probability of disappearance and reappearance of choices. It was shown that the inclusion of such information in behavioral models could favor IIA and transitivity violations in maximization of long-term energy gain^15^.

To facilitate a comparison to existing work on context-dependent preferences in animal and human literature, we framed our experimental and computational findings in terms of stochastic, discrete choice. In this framework, the choice of an individual worm is ascribed based on their ultimate proximity to one of the odors^43^. However, this framing should be viewed as a high-level synthesis of the different low-level behaviors that constitute the chemosensory decision process^24,44^. Rather than the true concentration of each odor option, individual worms encounter spatially varying concentration gradients. In turn, preference is expressed through a combination of behavioral strategies such as orthokinesis, klinotaxis, and klinokinesis that result in gradient ascent toward preferred odors. Thus, at the fine-grained level, the decision-making process is an ongoing, dynamic response to continuously changing stimuli. Given this continuous behavior, the discrete choice IIA framework and normalization model presented here should be viewed as a high-level description of average rather than instantaneous behavior. While not a description of microscopic behavior, this macroscopic framework is likely to be relevant for three reasons: first, the final outcomes realized by choice behavior play a large role in evolutionary fitness and were likely a target of natural selection; second, the lack of IIA violations in most scenarios we tested suggest that macroscopic rationality is a useful description of *C. elegans* decision-making; and finally, ongoing work suggests that even simple economic choice in monkeys and humans may reflect a dynamic and continuous process^45–47^.

Our experimental results and normalization-based mathematical model are aligned with previous studies on cortical computation which showed that IIA violations can be explained by a divisive normalization framework^5,48,49^. Thus, our work supports the notion that IIA violations are a result of neural constraints carved by evolution to maximize information under limited time and resources^50,51^, and directly relate to the high-level concept of “bounded rationality”^52^. We propose that understanding the building blocks of choice in an animal with a compact, deciphered, rigid, and stereotypic connectome can shed light on the fundamental biological constraints and principles that generate (non)rational behavior in simple as well as in complex organisms.

These findings and others establish a foundation to use *C. elegans* to investigate underlying neuronal mechanisms of decision making^23,44,53,54,55^.

## Methods

### Strains and husbandry

The strains used in this work: Bristol N2 wild-type, *cat-2* (n4547), and *nsy-1*(ky542). All strains were maintained at 20 °C on NGM plates supplemented with the antifungal agent Nystatin and fed with *E. coli* OP50^56^.

### Obtaining synchronized worms (“Egg-prep”)

A synchronized population of worms was obtained by employing a standard “egg-prep” procedure^56^.

NGM plates with gravid adults were washed into a 1.5 ml tube with M9 buffer. An egg-prep solution was prepared by mixing 7.5 ml of distilled water, 2 ml of household bleach (5% sodium hypochlorite solution) and 0.5 ml of 10 M NaOH solution. One millilitre of egg-prep solution was added to the tube containing the worms, and were shaken every minute for a total duration of 6 min. The tube was then centrifuged in 8000 RPM for 60 s, aspirated to 0.1 ml, and filled to the top with M9 buffer. This was repeated a total of three times. A Pasteur pipette was used to transfer the eggs to clean NGM plates seeded with *E. coli* OP50 bacteria.

### Chemotaxis assays

Chemotaxis assays were based on classical chemotaxis assays^24,32^. Unless stated otherwise, assay plates were square 12 × 12 cm dishes containing 30 ml of 1.6% BBL agar (Benton-Dickinson), 5 mM potassium phosphate (pH 6.0), 1 mM CaCI2 and 1 mM MgSO4. Assays in round plates were performed in 9 cm diameter dishes containing 12 ml of the mixture described above. Three marks were made on the back of the plates equidistant from the center of the plate (3 cm) and from each other (5.2 cm). The diluted attractants (1 µl) was placed on the agar over one marks. In the control plates (binary choice), 1 µl of 100% ethanol was placed over the third mark (all attractants were diluted in ethanol). The tested animals were placed at the center of the plate, equidistant from the three marks. Attractants were obtained from Sigma-Aldrich. Pure pyrazine is a solid, so pyrazine dilutions are weight:volume rather than volume:volume as for other attractants.

Well-fed adult animals were washed three times with wash buffer (0.5% Gelatin, 5 mM potassium phosphate (pH 6.0), 1 mM CaCI_2_ and 1 mM MgSO_4_), then placed near the center of a plate equidistant from the attractants (and the control spot when present). Approximately, 1 h after the assay began, the numbers of animals at the three areas (2 cm radius of each attractant) were determined, as well as the total number of animals in the assay, the number of animals that were not at any attractant area, and the number of animals that stayed in the starting point (did not cross a 1 cm diameter circle around the center of the plate). A specific C.I. was calculated as

$${\mathbf{A}}\,{\mathrm{Chemotaxis}}\,{\mathrm{Index}} = \frac{{{\mathrm{Number}}\,{\mathrm{of}}\,{\mathrm{animals}}\,{\mathrm{at}}\,{\mathrm{attractant}}\,{\mathbf{A}}}}{{{\mathrm{Number}}\,{\mathrm{of}}\,{\mathrm{animals}}\,{\mathrm{at}}\,{\mathrm{attractants}}\,{\mathbf{A}}\,{\mathrm{and}}\,{\mathbf{B}}}}$$

The C.I. can vary from 0 to 1. The animals were anaesthetized when they reached the attractant. One microlitre of sodium azide 1 M was placed at each one of the three spots, 15 min in advanced. Sodium azide anaesthetized animals within about a 1 cm radius of the attractant. For discrimination assays, acetone was added to a final concentration of 1.2 µl per 10-ml plate and mixed with the liquid agar once it had cooled to 55 °C, and the odor spots were placed 3.5 cm from the center. For assays where odor C is embedded in the agar, 2-butanone was added to a final concentration of 140 µl per 14-ml plate (dilution factor of 10^−2^) and mixed with the liquid agar once it had cooled to 50 °C and the odor spots were placed 3.5 cm from the center.

### “Bug” or “Feature” assays

We measured the relative preference between 2 µl of benzaldehyde (10^−2^) (A) and 2 µl of 2-butanone (10^−2^) (B), and compared it to the relative preference between 2 µl of benzaldehyde (10^−2^) (A), and a mixture of 1 µl of 2-butanone (1/50), and 1 µl of benzaldehyde (1/50) (A’B’). The 2-butanone spot (B) and the 2-butanone + benzaldehyde (A’B’) spot, contain the same amount of 2-butanone molecules, as well as an equal volume of ethanol. The “A’B’“ spot contains, in addition to 2-butanone, the same amount of benzaldehyde molecules as presented by “A”. Each assay included 3 “A vs. B” plates, coupled to 3 “A vs. A’B’“ plates. Each data point represents the mean of six essays performed on two different days.

### Statistical analysis

Data are presented as mean ± standard error of mean (SEM). Statistical significance of differences in chemotaxis index between control and test plates in a certain concentration were analyzed by a two-tailed Wilcoxon signed-ranks test. We corrected for multiple comparisons using the Benjamini–Hochberg false-discovery rate, with a false-discovery rate of 0.05. The *q* values reported in this study are adjusted to multiple comparisons (*q* < 0.05 was regarded as significant; *q < 0.05, **q < 0.01, ***q < 0.001, and ****q < 0.0001).

### Normalization model of sensory gain control

To examine whether both IIA and non-IIA choice behavior can be explained by a circuit-specific model of sensory gain control in chemosensation, we implemented a simple divisive normalization-based computational model of chemosensory value coding. Gain control is a widespread representational principle in early sensory processing in which the overall level of coding activity is regulated by the specific context present at the time of encoding. For example, Drosophila antennal lobe neural activity representing a specific odor will depend on whether other odors are present, and primate primary visual cortical responses to a center stimulus will be suppressed by stimuli in the sensory surround. Many of these gain control interactions can be explained a normalization computation, in which the feedforward-driven response of a neuron is divided by a term that represents a larger pool of neurons. This normalization pool (acting via the equation denominator) provides a mechanism for contextual modulation of the stimulus-specific response (in the numerator). For example, the response of Drosophila antennal lobe neurons is increased by a test odorant but suppressed by a mask odorant, a pattern described by normalization-based gain control.

To examine the predictions of a gain control model of chemosensation, we constructed a model of pathway-specific sensory gain control in *C. elegans* chemosensation and explored its qualitative predictions in trinary odorant choice behavior. In this model, neural activity *R*_*i*_ of a chemosensory neuron representing the decision value of an odorant stimulus *i* depends on its concentration (or intensity) *I*_*i*_ via a divisive normalization representation

$$R_i = \frac{{I_i}}{{\sigma + I_i}}{,}$$

where the semisaturation term *σ* controls how the function approaches saturation. Context-dependence in this model is instantiated as cross-odorant gain control when a given chemosensory neuron responds to more than one odor and both odors are present in the choice set. For example, in the most basic version of this model, the responses to two odors A and B will be described by the equations

$$R_A = \frac{{I_A}}{{\sigma + I_A + I_B}}$$

$$R_B = \frac{{I_B}}{{\sigma + I_A + I_B}}$$

where *I*_*A*_ and *I*_*B*_ are properties of the odor stimuli (i.e., concentrations) and the responses *R*_*A*_ and *R*_*B*_ denote neural activity representing the value of the odors. Note that this is an algorithmic model intended to model information processing rather than a biophysical implementation; however, because *C. elegans* neurons are generally thought to not exhibit action potentials, *R* can be viewed as a graded voltage signal. Furthermore, since this activity integrates across chemosensory neurons, it represents information at a downstream stage: synaptic input to interneurons, interneuron activity, or a more global measure of preference (e.g., turn/run balance in the klinokinesis-governing circuit).

In this model, odorants represented by multiple chemosensory neurons (e.g., benzaldehyde activating both AWC^ON^ and AWC^OFF^) receive a weighted averaging across the active neurons. For example, the response to benzaldehyde (denoted A here) in the presence of 2-butanone (AWC^ON^ only, here denoted B) is described as

$$R\left( A \right) = w_{\mathrm{ON}}\frac{{I_A}}{{\sigma + I_A + I_B}} + w_{\mathrm{OFF}}\frac{{I_A}}{{\sigma + I_A}}{,}$$

where the final response is a weighted sum of the responses of AWC^ON^ (left term) and AWC^OFF^ (right term). Note that for simplicity and parsimony, we assume that neurons that are encoding a single odor at a given time (AWC^OFF^ in the example above) also have an analogous form of gain control over the single represented odor. Decisions are implemented by a simple noisy decision rule, assuming a fixed Gaussian noise term (equal across all options in a choice set).

### Reporting summary

Further information on research design is available in the Nature Research Reporting Summary linked to this article.

## Supplementary information

Supplementary Information

Reporting Summary

## Data Availability

The datasets generated and analyzed in this study are available upon request to the corresponding author.

## Code availability

The dataset generated and analyzed in this study are available upon request to the corresponding author.

## References

[1] Afriat S. N. (1967). The Construction of Utility Functions from Expenditure Data. International Economic Review.

[2] Stearns SC (2000). Daniel Bernoulli (1738): evolution and economics under risk. J. Biosci..

[3] Kahneman D, Tversky A (1979). Prospect theory—analysis of decision under. Risk.

[4] Tversky A, Kahneman D (1992). Advances in prospect theory: cumulative representation of uncertainty. J. Risk Uncertain..

[5] Louie K, Khaw MW, Glimcher PW (2013). Normalization is a general neural mechanism for context-dependent decision making. Proc. Natl Acad. Sci. USA.

[6] Yamada H, Tymula A, Louie K, Glimcher PW (2013). Thirst-dependent risk preferences in monkeys identify a primitive form of wealth. Proc. Natl Acad. Sci. USA.

[7] Hurly TA, Oseen MD (1999). Context-dependent, risk-sensitive foraging preferences in wild rufous hummingbirds. Anim. Behav..

[8] Bateson M, Healy SD, Hurly TA (2003). Context-dependent foraging decisions in rufous hummingbirds. Proc. R. Soc. B Biol. Sci..

[9] Royle NJ, Lindström J, Metcalfe NB (2008). Context-dependent mate choice in relation to social composition in green swordtails *Xiphophorus helleri*. Behav. Ecol..

[10] Shafir S, Waite TA, Smith BH (2002). Context-dependent violations of rational choice in honeybees (*Apis mellifera*) and gray jays (*Perisoreus canadensis*). Behav. Ecol. Sociobiol..

[11] Shafir S (1994). Intransitivity of preferences in honey bees: support for ‘comparative’ evaluation of foraging options. Anim. Behav..

[12] Simon HA (1955). A behavioral model of rational choice. Q. J. Econ..

[13] Simon HA (1956). Rational choice and the structure of the environment. Psychol. Rev..

[14] Louie K, Glimcher PW (2012). Efficient coding and the neural representation of value. Ann. N. Y. Acad. Sci..

[15] JM M, PC T, AI H (2014). Natural selection can favour ‘irrational’ behaviour. Biol. Lett..

[16] Luce R. Duncan (2005). Individual choice behavior: A theoretical analysis.

[17] Louie K, Glimcher PW, Webb R (2015). Adaptive neural coding: from biological to behavioral decision-making. Curr. Opin. Behav. Sci..

[18] Tversky A, Simonson I (1993). Context-dependent preferences. Manag. Sci..

[19] Jarrell T. A., Wang Y., Bloniarz A. E., Brittin C. A., Xu M., Thomson J. N., Albertson D. G., Hall D. H., Emmons S. W. (2012). The Connectome of a Decision-Making Neural Network. Science.

[20] Borne F, Kasimatis KR, Phillips PC (2017). Quantifying male and female pheromone-based mate choice in Caenorhabditis nematodes using a novel microfluidic technique. PLoS ONE.

[21] Leighton DHW, Choe A, Wu SY, Sternberg PW (2014). Communication between oocytes and somatic cells regulates volatile pheromone production in *Caenorhabditis elegans*. Proc. Natl Acad. Sci. USA.

[22] White JQ (2007). The sensory circuitry for sexual attraction in *C. elegans* males. Curr. Biol..

[23] Barrios A (2014). Exploratory decisions of the *Caenorhabditis elegans* male: a conflict of two drives. Semin. Cell Dev. Biol..

[24] Bargmann CI, Hartwieg E, Horvitz HR (1993). Odorant-selective genes and neurons mediate olfaction in *C. elegans*. Cell.

[25] Bargmann, C. I. Chemosensation in *C. elegans*. *WormBook* (WormBook, 2006). 10.1895/wormbook.1.123.1 10.1895/wormbook.1.123.1 PMC4781564 18050433

[26] Worthy SE, Rojas GL, Taylor CJ, Glater EE (2018). Identification of odor blend used by *Caenorhabditis elegans* for pathogen recognition. Chem. Senses.

[27] Choi JI (2018). Odor-dependent temporal dynamics in *Caenorhabitis elegans* adaptation and aversive learning behavior. PeerJ.

[28] Wes PD, Bargmann CI (2001). *C. elegans* odour discrimination requires asymmetric diversity in olfactory neurons. Nature.

[29] Alqadah, A., Hsieh, Y., Xiong, R. & Chuang, C. *Stochastic Left—Right Neuronal Asymmetry in Caenorhabditis elegans* (2016). 10.1098/rstb.2015.0407 PMC5104506 27821536

[30] Von Neumann, J. & Morgenstern, O. *Theory of Games and Economic Behavior.* (Princeton University Press, 1944).

[31] Samuelson PA (1938). A note on the pure theory of consumer’s behaviour. Economica.

[32] Ward S (1973). Chemotaxis by the nematode *Caenorhabditis elegans*: identification of attractants and analysis of the response by use of mutants. Proc. Natl Acad. Sci. USA.

[33] Doya K (2008). Modulators of decision making. Nat. Neurosci..

[34] Rogers RD (2011). The roles of dopamine and serotonin in decision making: evidence from pharmacological experiments in humans. Neuropsychopharmacology.

[35] Dennis EJ (2018). A natural variant and engineered mutation in a GPCR promote DEET resistance in C. elegans. Nature.

[36] Sagasti A, Hobert O, Troemel ER, Ruvkun G, Bargmann CI (1999). Alternative olfactory neuron fates are specified by the LIM homeobox gene lim-4. Genes Dev..

[37] Schulenburg H, Félix M-A (2017). The natural biotic environment of *Caenorhabditis elegans*. Genetics.

[38] Hodgkin, J. What does a worm want with 20,000 genes? *Genome Biol*. **2**, COMMENT2008 (2001). 10.1186/gb-2001-2-11-comment2008 PMC138976 11737938

[39] Axel R (2005). Scents and sensibility: a molecular logic of olfactory perception (Nobel Lecture). Angew. Chem. Int. Ed..

[40] Buck L, Axel R (1991). A novel multigene family may encode odorant receptors: a molecular basis for odor recognition. Cell.

[41] Bargmann CI, Kaplan JM (1998). Signal transduction in the *Caenorhabditis elegans* nervous system. Annu. Rev. Neurosci..

[42] Nickell WT, Pun RYK, Bargmann CI, Kleene SJ (2002). Single ionic channels of two *Caenorhabditis elegans* chemosensory neurons in native membrane. J. Membr. Biol..

[43] Hart, A. C. Behavior. in *WormBook* (ed. The C. elegans Research Community) (WormBook, 2006). 10.1895/wormbook.1.87.1

[44] Faumont S, Lindsay T, Lockery S (2012). Neuronal microcircuits for decision making in *C. elegans*. Curr. Opin. Neurobiol..

[45] Krajbich I, Rangel A (2011). Multialternative drift-diffusion model predicts the relationship between visual fixations and choice in value-based decisions. Proc. Natl Acad. Sci..

[46] Rich EL, Wallis JD (2016). Decoding subjective decisions from orbitofrontal cortex. Nat. Neurosci..

[47] Shadlen MN, Shohamy D (2016). Decision making and sequential sampling from memory. Neuron.

[48] Webb, R., Glimcher, P. W. & Louie, K. Rationalizing context-dependent preferences: divisive normalization and neurobiological constraints on choice. *SSRN Electron. J*. 10.2139/ssrn.2462895 (2014).

[49] Louie K, Grattan LE, Glimcher PW (2011). Reward value-based gain control: divisive normalization in parietal cortex. J. Neurosci..

[50] Cochella L (2014). Two distinct types of neuronal asymmetries are controlled by the *Caenorhabditis elegans* zinc finger transcription factor die-1. Genes Dev..

[51] Palmer AR (1996). From symmetry to asymmetry: phylogenetic patterns of asymmetry variation in animals and their evolutionary significance. Proc. Natl Acad. Sci. USA.

[52] Simon, H. Theories of Bounded Rationality. in Decision and Organization (eds. McGuire, C. B. & Radner, R.) 161–176 (North-Holland Pub. Co, 1972).

[53] Iwanir S (2019). Irrational behavior in *C. elegans* arises from asymmetric modulatory effects within single sensory neurons. Nat. Commun..

[54] Bendesky A, Tsunozaki M, Rockman MV, Kruglyak L, Bargmann CI (2011). Catecholamine receptor polymorphisms affect decision-making in C. elegans. Nature.

[55] Ghosh D (2016). Neural Architecture of Hunger-Dependent Multisensory Decision Making in C. elegans. Neuron.

[56] Stiernagle, T. *Maintenance of C. elegans* (WormBook, 2006). 10.1895/wormbook.1.101.1 10.1895/wormbook.1.101.1 PMC4781397 18050451

