# Supplementary material for: Bounded rationality in C. elegans is explained by circuit-specific normalization in chemosensory pathways
Source: Nat Commun. 2019 Aug 13;10:3692. doi: 10.1038/s41467-019-11715-7 (PMC6692327; doi:10.1038/s41467-019-11715-7)
Supplement: Supplementary file 1 — Supplementary Information [file 41467_2019_11715_MOESM1_ESM.pdf]

Supplementary Materials for

Bounded rationality in *C. elegans* is explained by circuit-specific normalization in chemosensory pathways

Dror Cohen[$], Guy Teichman[$], Meshi Volovich, Yoav Zeevi, Lilach Elbaum, Asaf Madar, Kenway Louie, Dino J Levy[§,*], Oded Rechavi[§,*]

$ These authors contributed equally.

§ These authors jointly supervised this work.

*Corresponding authors: dinolevy@post.tau.ac.il&odedrechavi@gmail.com

This PDF file includes:

Figs. S1 to S12

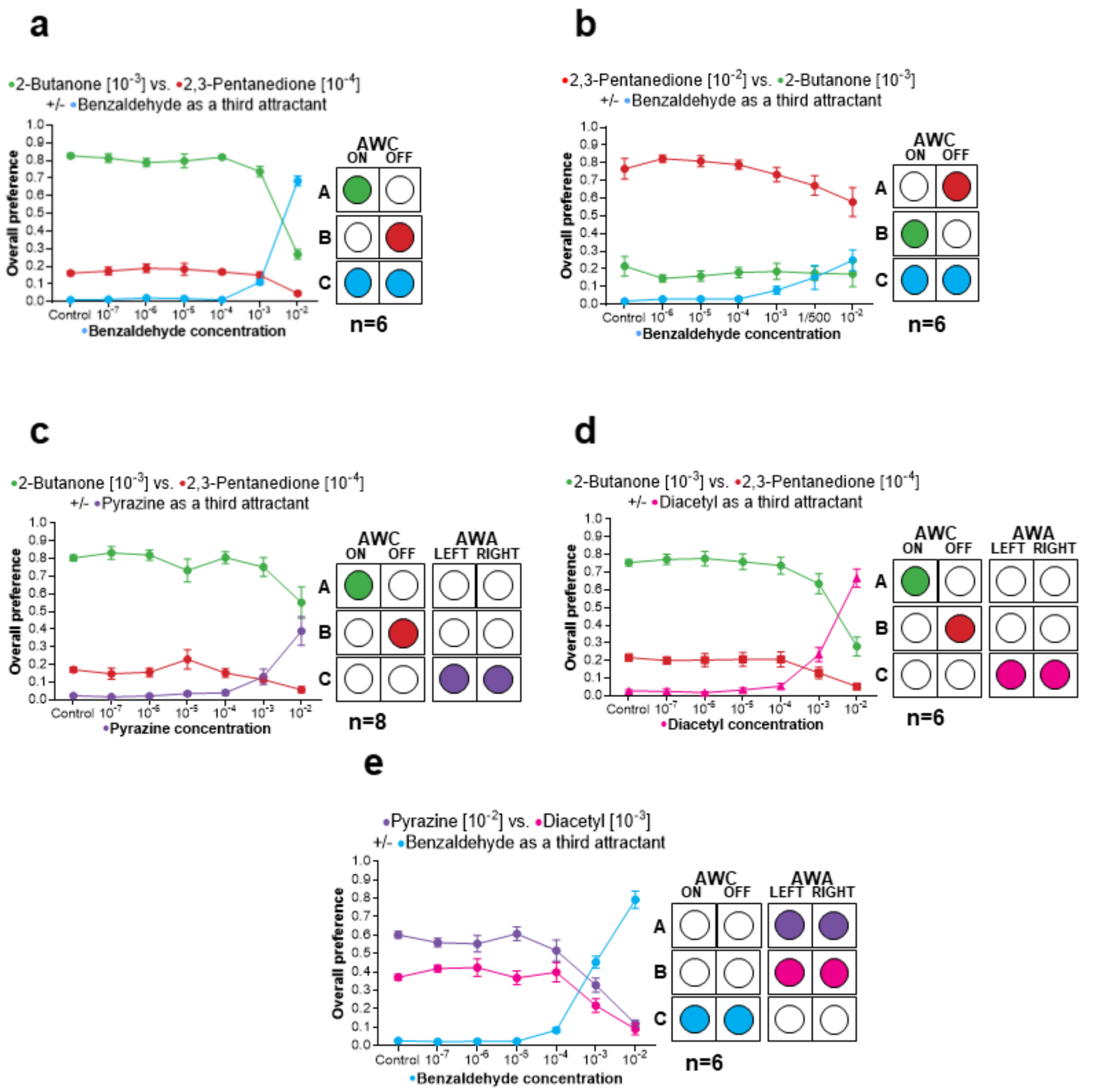

Fig. S1. *C. elegans* display rational decisions – overall preferences. Overall preferences (number of worms arrived at an odor spot, divided by the total number of worms on the assay plate) in the two experiments described in **Fig.1, b-f.**

(a) The relative preference for 2-butanone (10^{-3}) over 2,3-pentanedione (10^{-4}) is unaffected by increasing concentration of benzaldehyde as a third attractant. (**b)** The relative preference for 2,3-pentanedione (10^{-2}) over 2-butanone (10^{-3}) is unaffected by increasing concentration of benzaldehyde as a third attractant. **(c-d**) introducing AWA sensed odorants as a third attractant, does not influence the relative preference between 2-butanone (10^{-3}) and 2,3-pentanedione (10^{-4}). **(e)** benzaldehyde as a third attractant, does not affect the relative preference between the two AWA sensed odorants pyrazine (10^{-2}) and diacetyl (10^{-3}). Error bars represent the standard error of the mean C.I.

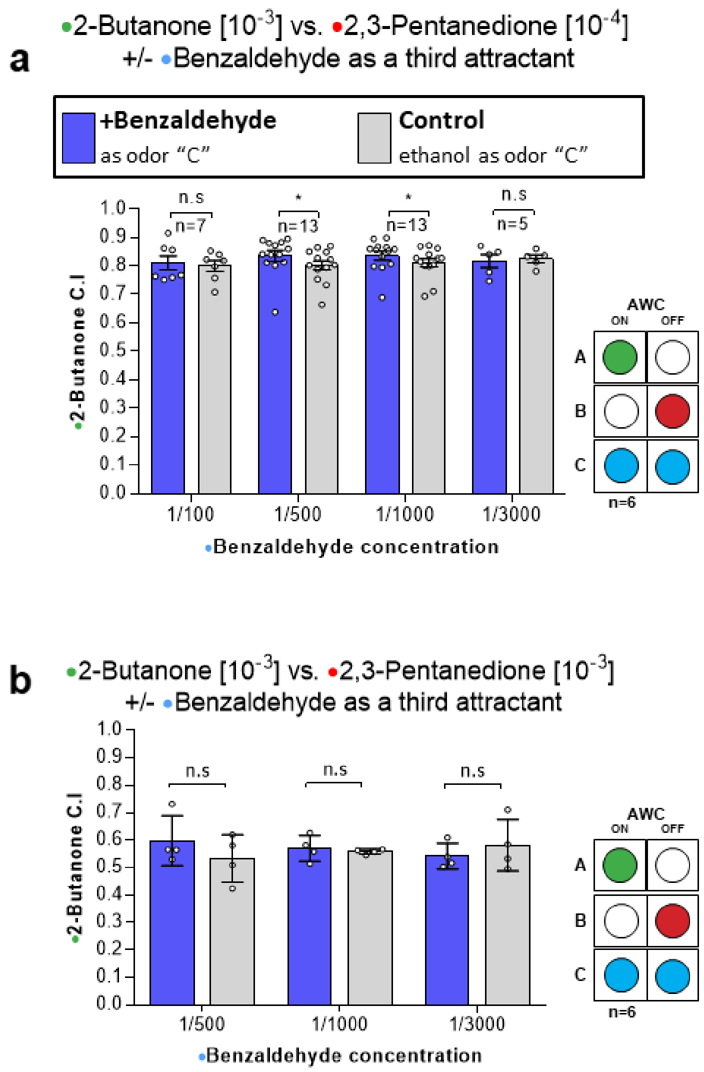

Fig. S2. Benzaldehyde (AWC^{BOTH}) as a third attractant does not change the relative preference between 2-butanone (AWC^{ON}) and 2,3-pentanedione (AWC^{OFF}), in wild-type mutants. Benzaldehyde as a third attractant does not influence the relative preference between **(a)** 2-butanone (10^{-3}) and 2,3-pentanedion (10^{-4}) (Wilcoxon Signed-Ranks Test, C=10^{-2}: W=332, q=0.4893; C=1/500: W=1395, q=0.012; C=10^{-3}: W=1280, q=0.034; C=1/3000: W=82, q=0.495) and **(b)** 2-butanone (10^{-3}) and 2,3-pentanedion (10^{-3}) (Wilcoxon Signed-Ranks Test, C=1/500: W=175, q=0.501; C=1/1000: W=141, q=0.642; C=1/3000: W=96, q=0.642; n=4). The very weak differences that were observed here were considered physiologically irrelevant. Each replication consisted of 3 experimental plates and 3 control plates, for each concentration of odor C.

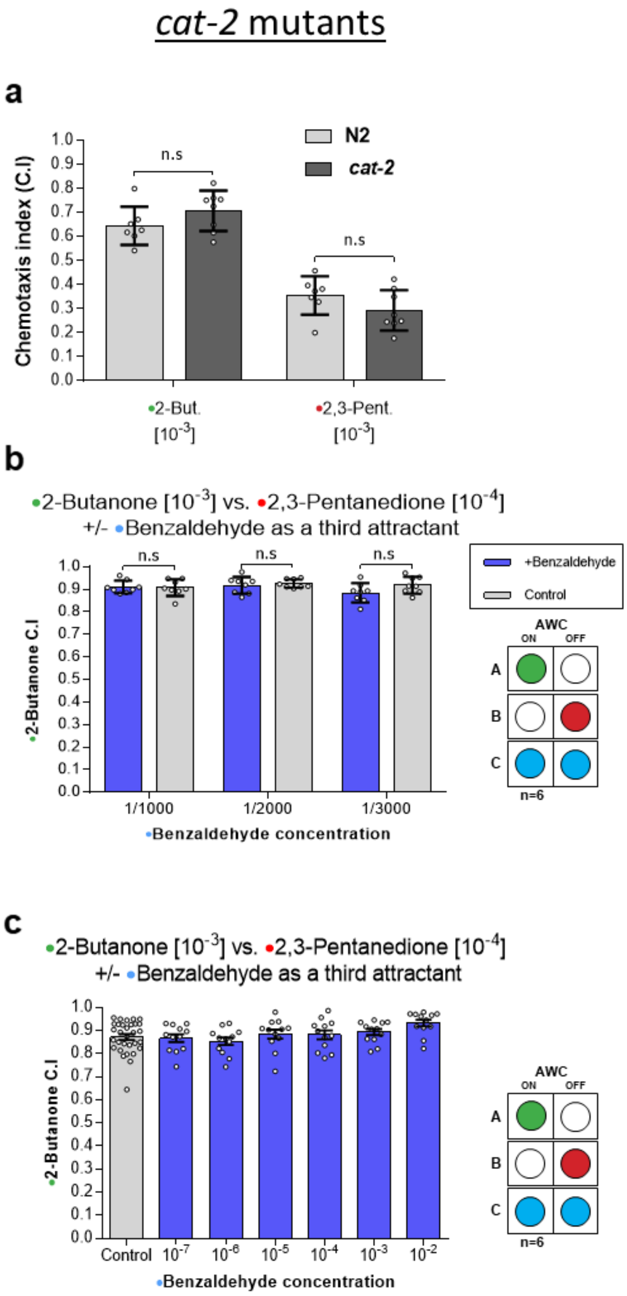

Fig. S3. The introduction of the benzaldehyde as a third attractant did not have any significant effect on the preference between 2-butanone and 2,3-pentanedione in *cat-2* mutants.

The *cat-2* gene encodes for tyrosine hydroxylase, the rate limiting enzyme in the synthesis of dopamine. **(a)** There are no significant differences between wild-type (N2) and *cat-2* mutants in their relative preferences between 2-butanone (10^{-3}) and 2,3-pentanedion (10^{-4}) (Wilcoxon Signed-Ranks Test, 2-butanone: W=38 ,q=0.28 ; 2,3-pentanedione: W=18 , q=0.28 ; n=8). Error bars

represent standard deviation. **(b)** The effect of benzaldehyde as a third attractant on the relative preference between 2-butanone (10^{-3}) and 2,3-pentanedion (10^{-4}) in *cat-2* mutants (Wilcoxon Signed-Ranks Test, C=1/500: W=175, q=0.501; C=10^{-3}: W=141, q=0.642; C=10^{-3}/3: W=96 , q=0.642; n=8). **(c)** *cat-2* mutants' relative preference between 2-butanone (10^{-3}) and 2,3-pentanedion (10^{-4}) is unaffected by the introduction of benzaldehyde as a third attractant (Wilcoxon Signed-Ranks Test, C=10^{-7}: W=172, q=0.9967; C=10^{-6}: W=148, q=0.9967; C=10^{-5}: W=206, q=0.9967; C=10^{-4}: W=193, q=0.9967; C=10^{-3}: W=155, q=0.9967; C=10^{-2}: W=80, q=0.9967; n=12). Bars represent the mean C.I of 2-butanone. Wilcoxon Signed-Ranks Tests, Error bars represent standard error of the mean C.I.

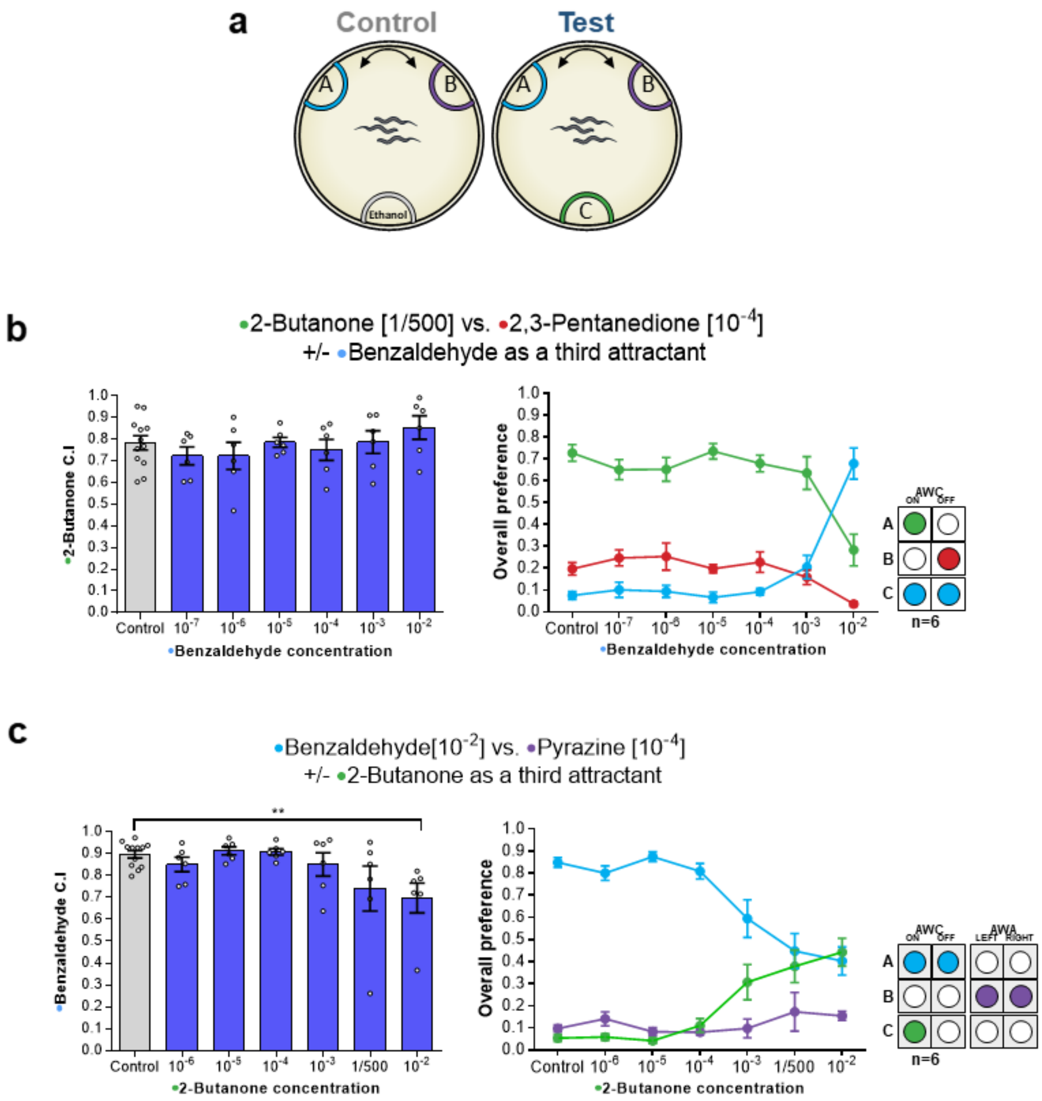

Fig. S4. The size and symmetry of agar plates do not affect the occurrence of IIA violations.

Worms on round plates with a smaller radius and smaller odor spot size behave similarly to worms on larger square plates. **(a)** A scheme of the experiments performed on round plates. **(b)** The relative preference for 2-butanone (1/500) over 2,3-pentanedione (10^{-4}) is unaffected by increasing concentration of benzaldehyde as a third attractant (Wilcoxon Signed-Ranks Test, $C=10^{-7}$: W=25, q=0.9636; $C=10^{-6}$: W=28, q=0.9636; $C=10^{-5}$: W=35, q=0.9636; $C=10^{-4}$: W=32, q=0.9636; $C=10^{-3}$: W=35, q=0.9636; $C=10^{-2}$: W=24, q=0.9636; n=6). **(c)** The presence of 2-butanone as a third attractant significantly reduced the relative preference for benzaldehyde (10^{-2}) over pyrazine (10^{-4}) (Wilcoxon Signed-Ranks Test, $C=10^{-6}$: W=24, q=0.5816; C=10^{-}

^{5}: W=28, q=0.7404; C=10^{-4}: W=35, q=0.9636; C=10^{-3}: W=35, q=0.9636; C=1/500: W=19, q=0.3738; C=10^{-2}: W=1, q=0.0012; n=6).

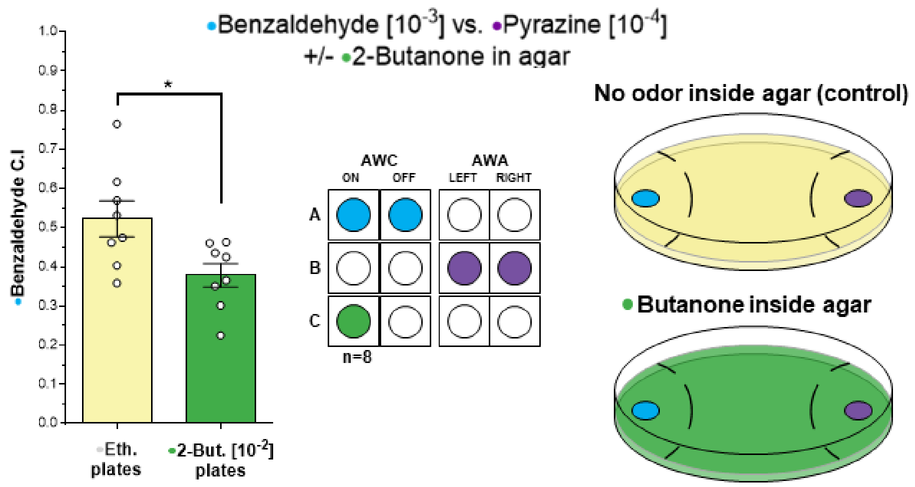

Fig. S5. 2-butanone (AWC^{ON}) mixed into the agar disproportionally reduces the preference for benzaldehyde over pyrazine.

A binary preference between benzaldehyde (10^{-3}) and pyrazine (10^{-4}) which was performed either on regular agar plates, or on plates which contain 2-butanone (10^{-2}) (see Methods) (Wilcoxon Signed-Ranks Test, W=10, p=0.0207, n=8). Bar represent chemotaxis index of benzaldehyde, on a 2-butanone agar plate (green) and on a plain agar plate (yellow). Error bars represent standard error of the mean C.I.

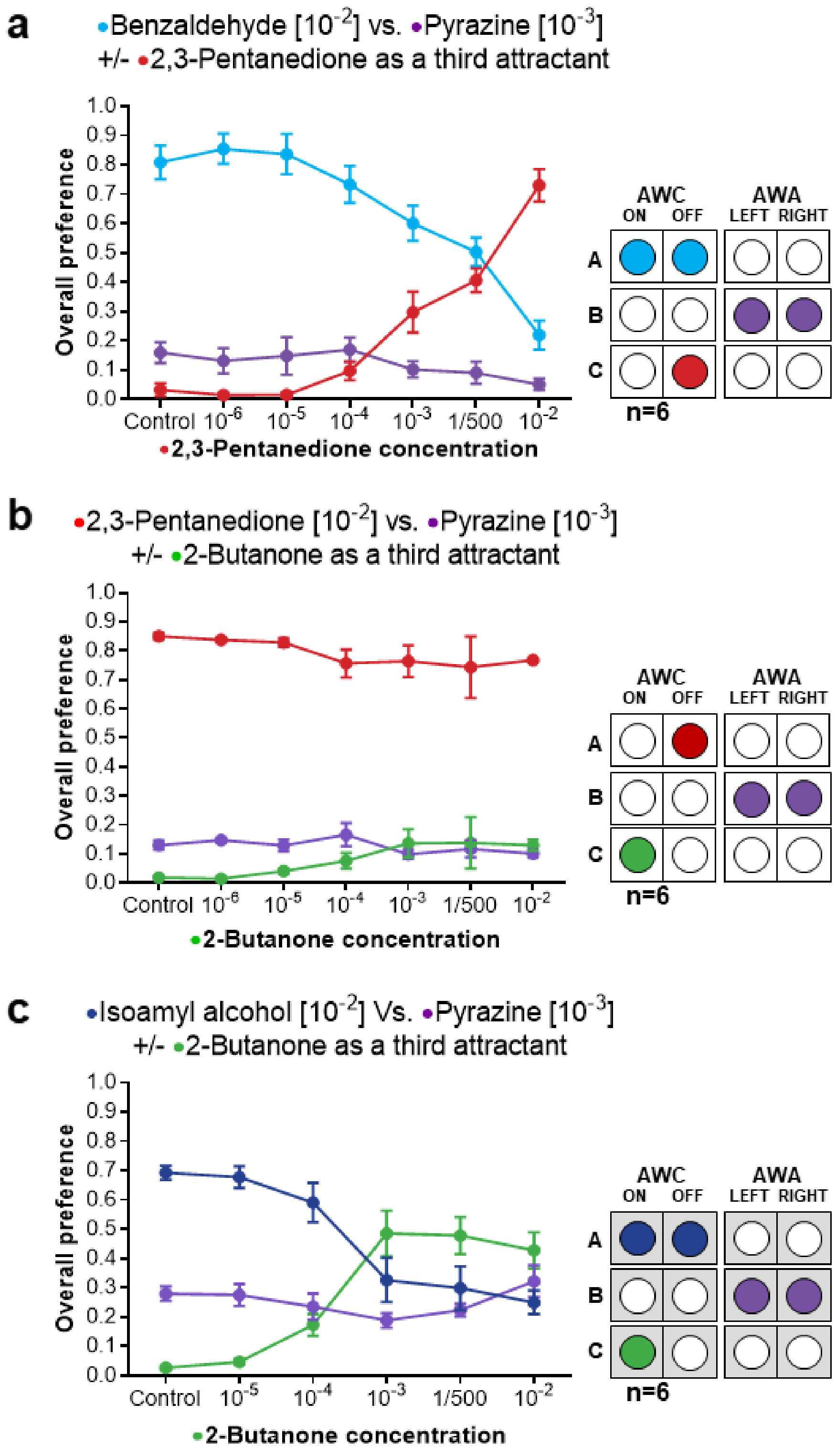

Fig. S6. *C. elegans* exhibit IIA violations when specific neuronal architectures are induced – overall preferences.

Overall preferences (number of worms arrived at an odor spot, divided by the total number of worms on the assay plate) in the two experiments described in **Fig.2, d-f**.

(a) 2,3-pentanedione as a third attractant does not change the relative preference between benzaldehyde (10^{-2}) and pyrazine (10^{-3})**.** **(b)** 2-butanone as a third attractant does not change the relative preference between 2,3-pentanedione (10-^{2}) and pyrazine (10^{-3}). **(c)** 2-butanone as a third attractant significantly reduced the relative preference for isoamyl-alcohol (10^{-2}) over pyrazine (10^{-3}). Error bars represent the standard error of the mean C.I.

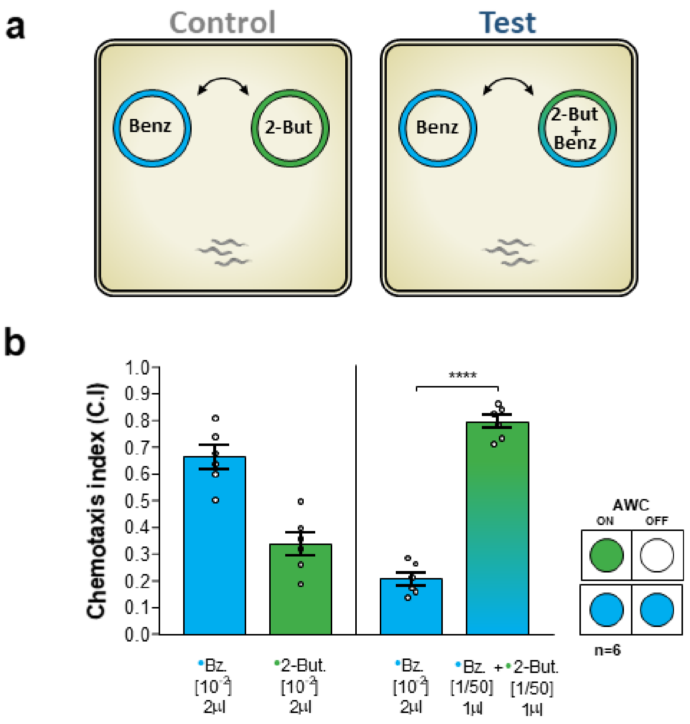

Fig. S7. 2-butanone and benzaldehyde together are as attractive as would be expected based on the simple summation of the attractiveness of each of the odors alone. A binary preference between benzaldehyde and a mixed combination of benzaldehyde and 2-butanone (placed in the same spot on the plate, see methods). We found that the combination of 2-butanone and benzaldehyde was more attractive than benzaldehyde alone (Wilcoxon Signed-Ranks Test, W=16.5, p=0.0000; n=6).

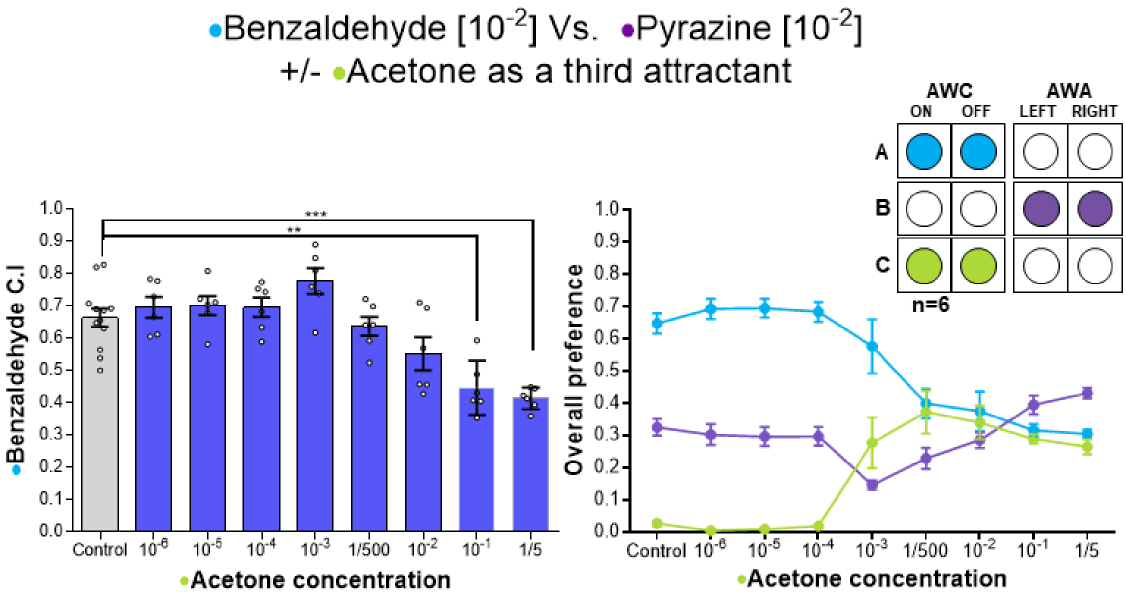

Fig. S8. Increasing concentrations of acetone (AWC^{ON}-sensed odor) as a third alternative can induce IIA violations and preference reversal.

The influence of acetone (AWC^{ON}) as a third attractant on the relative preference between benzaldehyde (10^{-2}) (AWC^{BOTH}) and pyrazine (10^{-3}) (AWA) (Wilcoxon Signed-Ranks Test, $C=10^{-6}$: W=30, q=0.6820; $C=10^{-5}$: W=24, q=0.3877; $C=10^{-4}$: W=29, q=0.6820; $C=10^{-3}$: W=15, q=0.1411; C=1/500: W=31, q=0.6820; $C=10^{-2}$: W=22, q=0.4258; $C=10^{-1}$: W=3, q=0.0032; C=1/5: W=0, q=0.0008; n=6). Bars represent the C.I of odor A. Error bars represent the standard error of the mean C.I.

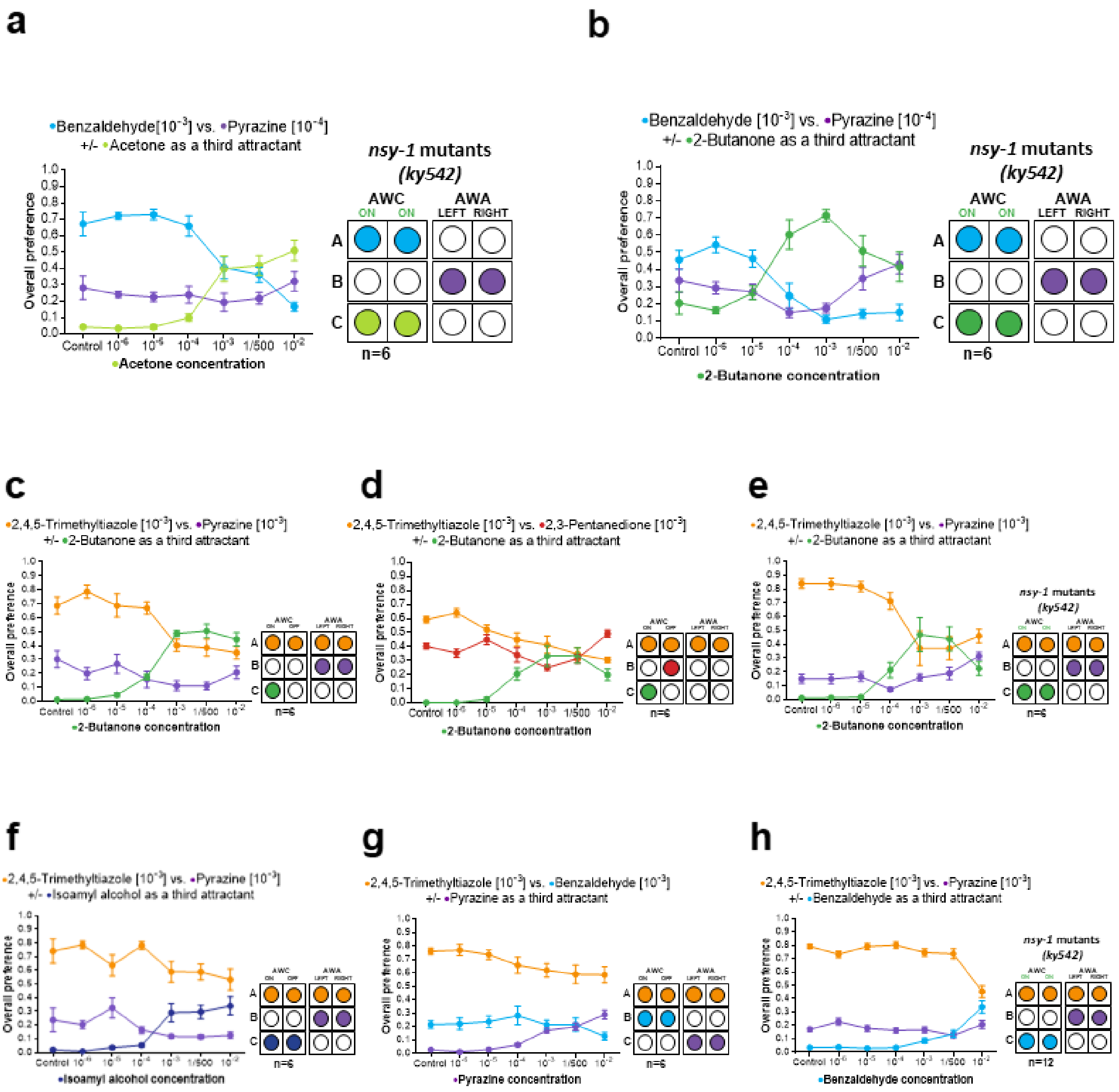

Fig. S9. The AWC^{ON} neuron makes the worm vulnerable to IIA violations - Overall preferences.

Overall preferences (number of worms arrived at an odor spot, divided by the total number of worms on the assay plate) in the two experiments described in **Fig.3 a-h.**

(a-b) AWC$^{ON/ON}$ mutants are hypersensitive to acetone and butanone, since they sense these odors using two AWC^{ON} neurons instead of one; therefore, in relatively low concentrations, these odors instantly became the most attractive odors on the plate**.** **(c)** 2-butanone as a third attractant does not change the relative preference between 2,4,5-trimethylthiazole (10^{-3}) and pyrazine (10^{-3}) (Wilcoxon Signed-Ranks Test, n=6). **(d)** The influence of 2-butanone as a third attractant on the relative preference between 2,4,5-trimethylthiazole (10^{-3}) and

2,3-pentanedione (10^{-3}). **(e)** 2-butanone as a third attractant changes the relative preference between 2,4,5-trimethylthiazole (10^{-3}) and pyrazine (10^{-3}) in AWC^{ON}/^{ON} mutant worms. **(f)** Isoamyl-alcohol as a third attractant does not change the relative preference between 2,4,5-trimethylthiazole (10^{-3}) and pyrazine (10^{-3}). **(g)** Pyrazine as a third attractant does not change the relative preference between 2,4,5-trimethylthiazole (10^{-3}) and benzaldehyde (10^{-3}). **(h)** Benzaldehyde as a third attractant changes the relative preference between 2,4,5-trimethylthiazole (10^{-3}) and pyrazine (10^{-3}). Error bars represent the standard error of the mean C.I.

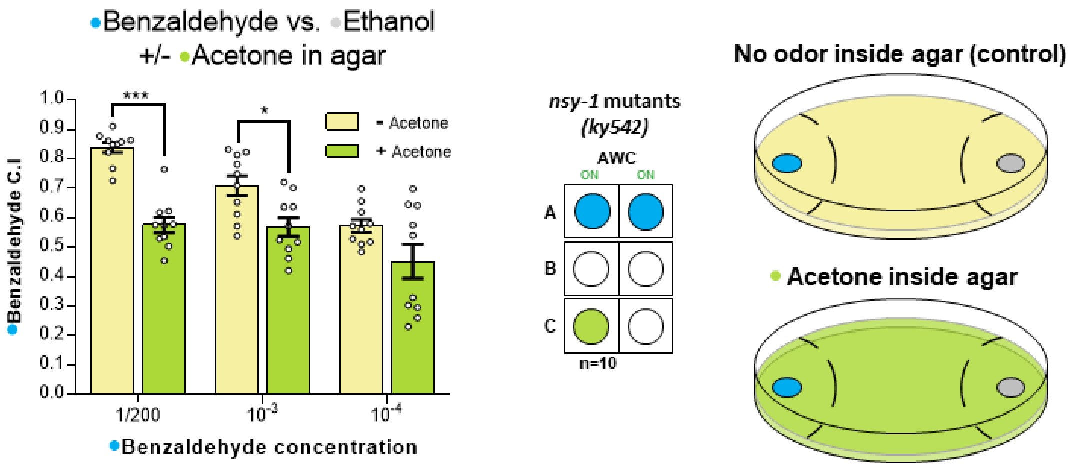

Fig. S10. ***nsy-1(ky542)*** **mutants (AWC$^{ON/ON}$ phenotype) fail to detect benzaldehyde (AWC^{BOTH}) when acetone (AWC^{ON}) is mixed into the agar.**

A binary preference between benzaldehyde and ethanol which was performed either on regular agar plates, or on plates which contain acetone (10^{-2}) (see Methods) (Wilcoxon Signed-Ranks Test, C=1/200: W=1, q<0.0003, n=10; C=10^{-3} : W=11, q<0.018, n=10; C=10^{-4} : W=35, q<0.28, n=10). Bar represent chemotaxis index of benzaldehyde, on an acetone agar plate (green) and on a plain agar plate (yellow). Error bars represent standard error of the mean C.I.

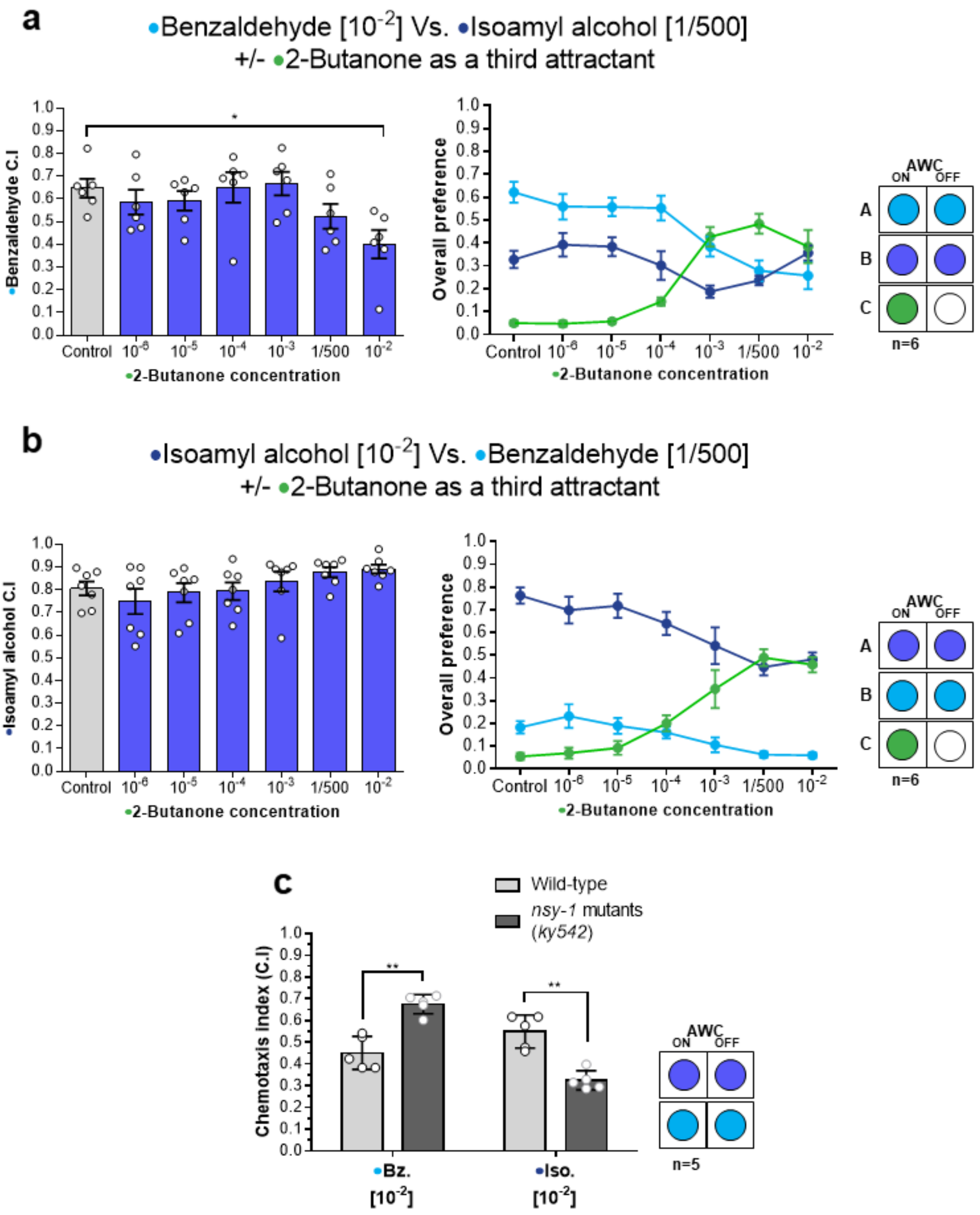

Fig. S11. AWC^{ON} is more important for sensation of benzaldehyde (AWC^{BOTH}) than for isoamyl alcohol (AWC^{BOTH}).

(a) The presence of 2-butanone as a third attractant significantly reduces the relative preference for benzaldehyde (10^{-2}) over isoamyl alcohol (1/500). (Wilcoxon Signed-Ranks Test, $C=10^{-6}$: W=11, q=0.4642; $C=10^{-5}$: W=16, q=0.8182; $C=10^{-4}$: W=11, q=0.4642; $C=10^{-3}$: W=14, q=0.7064; C=1/500: W=9, q=0.4642; $C=10^{-2}$: W=1, q=0.0258; n=6). **(b)** The presence of 2-butanone as a third attractant does not affect the relative preference for isoamyl alcohol (10^{-2}) over benzaldehyde (1/500). (Wilcoxon Signed-Ranks Test, $C=10^{-6}$: W=21,

q=0.9015; C=10^{-5}: W=22, q=0.9015; C=10^{-4}: W=23, q=0.9015; C=10^{-3}: W=15, q=0.5186; C=1/500: W=10, q=0.2184; C=10^{-2}: W=10, q=0.2184; n=6). **(c)** A binary preference between benzaldehyde (10^{-2}) and isoamyl alcohol (10^{-2}), in wild-type nematodes and in AWC$^{ON/ON}$ mutants. AWC$^{ON/ON}$ mutants show a significantly higher preference for benzaldehyde over isoamyl alcohol in comparison to wild-type worms' preference (Wilcoxon Signed-Ranks Test, W=0, p=0.0079; n=5).

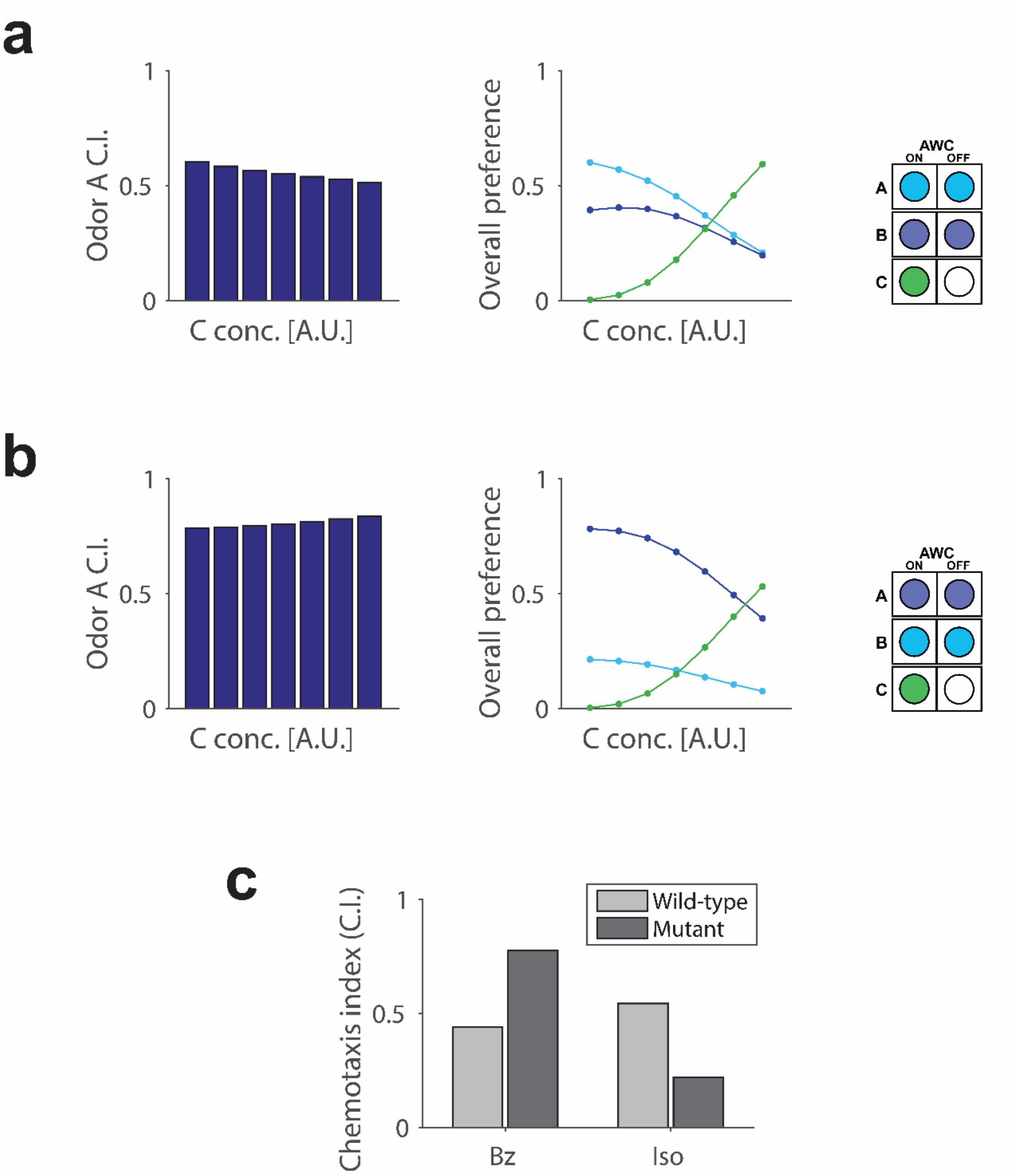

Fig. S12. The normalization model captures relative importance of AWC^{ON} neuron via differential weighting of chemosensory neuron output across odors.

Model predictions were made for choice scenario involving benzaldehyde (light blue), isoamyl alcohol (dark blue), and butanone (green). The model assumes cross-odor normalization in both AWC^{ON} and AWC^{OFF} neurons, but a biased weighting of chemosensory neuron output for benzaldehyde and an unbiased weighting for isoamyl alcohol. The same model parameters were used for all data in the figure. (a) Relative preference for benzaldehyde versus

isoamyl alcohol decreases as a function of increasing odor C (butanone) concentrations. (b) Relative preference for isoamyl alcohol versus benzaldehyde increases as a function of increasing odor C (butanone) concentrations. (c) Binary preference between benzaldehyde and isoamyl alcohol differs between wild-type and $AWC^{ON/ON}$ mutants.
